# Foliar fungi of *Betula pendula*: impact of tree species mixtures and assessment methods

**DOI:** 10.1038/srep41801

**Published:** 2017-02-02

**Authors:** Diem Nguyen, Johanna Boberg, Michelle Cleary, Helge Bruelheide, Lydia Hönig, Julia Koricheva, Jan Stenlid

**Affiliations:** 1 Department of Forest Mycology and Plant Pathology, Swedish University of Agricultural Sciences, 75007 Uppsala, Sweden; 2 Department of Organismal Biology, Uppsala University, 75236 Uppsala, Sweden; 3 Southern Swedish Forest Research Centre, Swedish University of Agricultural Sciences, 23053 Alnarp, Sweden; 4 Institute of Biology/Geobotany and Botanical Garden, Martin Luther University Halle-Wittenberg, 06108 Halle, Germany; 5 German Centre for Integrative Biodiversity Research (iDiv) Halle-Jena-Leipzig, 04103 Leipzig, Germany; 6 Royal Holloway University of London, Egham, Surrey TW20 0EX, United Kingdom

## Abstract

Foliar fungi of silver birch (*Betula pendula*) in an experimental Finnish forest were investigated across a gradient of tree species richness using molecular high-throughput sequencing and visual macroscopic assessment. We hypothesized that the molecular approach detects more fungal taxa than visual assessment, and that there is a relationship among the most common fungal taxa detected by both techniques. Furthermore, we hypothesized that the fungal community composition, diversity, and distribution patterns are affected by changes in tree diversity. Sequencing revealed greater diversity of fungi on birch leaves than the visual assessment method. One species showed a linear relationship between the methods. Species-specific variation in fungal community composition could be partially explained by tree diversity, though overall fungal diversity was not affected by tree diversity. Analysis of specific fungal taxa indicated tree diversity effects at the local neighbourhood scale, where the proportion of birch among neighbouring trees varied, but not at the plot scale. In conclusion, both methods may be used to determine tree diversity effects on the foliar fungal community. However, high-throughput sequencing provided higher resolution of the fungal community, while the visual macroscopic assessment detected functionally active fungal species.

Biodiversity is considered to be important for ecosystem services and functions^1^,^2^. Forests provide, among other things, timber and pulp, carbon sequestration, and habitats for plants and animals that, by extension, benefit humans. Tree species diversity has been linked to increases in forest ecosystem services and functions^3^,^4^. Furthermore, mixed forest stands are generally considered to be better adapted to changing environmental conditions, and are more resistant to abiotic and biotic damages than pure species stands^5^,^6^. Diverse forests can contribute to reduced susceptibility of trees to disease and pathogen infection, and a subsequent increase in plant survival and growth^7^,^8^. Despite increasing knowledge of tree species diversity effects on fungal pathogens, it is not yet clear how mixed forest stands influence foliar fungal communities^9^,^10^,^11^.

Tree leaves support a variety of microorganisms across broad taxonomic groups. The foliar community includes fungi with differing ecological strategies, such as endophytes, saprotrophs, and pathogens^12^. The diversity of these foliar fungi can be affected by the composition of neighbouring tree species in mixed forests. Tree species composition shapes the biotic and abiotic environment in which the fungi live, reproduce, disperse and eventually infect new hosts to begin the cycle again.

Several ecological processes that apply to fungal pathogens may explain tree species diversity effects on fungal diversity and composition. The interaction between a pathogen and an individual plant is influenced by the tree neighbourhood context of that plant^13^. Non-host trees can reduce disease risks when the density of these heterospecific trees increases; the proportion of host trees thus becomes diluted in mixed stands^7^. As a result of fewer available host trees and an increased distance between host species, tree species diversity can negatively impact fungal pathogens that specialize on a specific host^14^,^15^. On the other hand, generalist pathogens, whose resource specialization is broad, may be less affected by tree species diversity^16^,^17^. The spread of fungal pathogens can be limited by the presence of non-susceptible host species admixed with susceptible hosts^18^. Non-host species have also been suggested to promote natural enemies that can limit or prevent pathogen infection, or become barriers to disease spread^14^,^19^,^20^,^21^. The diversity of tree species in the immediate surrounding of a focal tree, i.e., at the scale of the local neighbourhood, can also have an impact on fungal species richness and pathogen infestation. A focal tree either experiences competition or facilitation by its neighbouring trees, and as such, can influence the ability of the focal tree to respond to the pathogen. Hantsch, *et al*. showed that pathogen infestation decreased on important forestry tree species when the focal trees were in the context of high local tree diversity^9^. A potential mechanism for the observed effects may be the alteration of the microclimate that emerged from mixing tree species, which may influence the biology of the pathogen^9^,^22^. Lastly, the presence of specific tree species, resulting in tree species identity effects, rather than the diversity of tree species, has been shown to influence fungal infections^10^,^22^,^23^. For example, when *Quercus* was present in a plot with other tree species, pathogen richness and load was found to be higher in those plots^10^. The above-mentioned ecological processes have thus far been applied to pathogens. Whether these processes extend to the composition and distribution of the complete foliar fungal community is still unknown.

For a long time, traditional culture-dependent methods have been used to discover, detect, and identify fungal species associated with leaves. Within a single leaf, a diversity of fungi may be observed^24^. However, only a fraction of all fungi present can be isolated and cultivated, which typically are the faster growing and the more dominant species of the fungal community^25^. Slower growing fungal species that could be important members of the community or those for which laboratory growth conditions have not yet been determined, may therefore be grossly underrepresented and will lead to a skewed representation of the fungal community. This called for the development of additional identification techniques. Recent ecological research has utilized visual morphological assessment and molecular methods, often employed independently due to technical constraints and/or lack of expertise, to study the fungal communities of leaves^9^,^26^.

Visual examination of fungal fruiting structures or damage symptoms can help associate these attributes with previously characterized species and quantify the amount of damage^9^. Furthermore, fungi that make recognizable structures or characteristic damages can be identified to genus level, and in some cases to species level, depending on the experience of the observer. Unfortunately, many species that may be present in or on the leaves make no observable signs or symptoms, or have a long latency period before fruiting becomes evident^27^, and thus fail to be detected. Another problem is that the fungi may simply have not yet been described at all. Despite these limitations, Hantsch and colleagues utilized visual detection methods of fungal taxa on leaves and found no significant tree species richness effects on foliar fungal species richness and pathogen load^10^.

Molecular methods have aided in the determination of fungal taxa richness, e.g. through sequencing of the internal transcribed spacer (ITS) regions of the ribosomal RNA genes^28^,^29^. Recent technological advances in next generation high-throughput sequencing methods, such as 454 pyrosequencing, have lead to the detection of fungi from a variety of complex environmental sources such as forest soil and plant tissue^30^. Beyond describing the fungal taxa present in a given environment, high-throughput sequencing methods have enabled further studies to understand ecological phenomena. For example, vegetation zones, warming, and host genotypes were found to influence fungal community composition, diversity, and function^31^,^32^,^33^.

Visual macroscopic assessment and molecular high-throughput sequencing approaches are expected to show the same response to tree species diversity, unless: a) the molecular approach fails to detect morphologically visible species. This may occur if, for example, DNA from all organisms present cannot be fully extracted or has been degraded, and/or b) the quantification differs (e.g., sequence reads vs. leaf area covered), which may result from damage being lower or higher than the amount of fungal DNA extracted. Both the visual and molecular methods are currently used independently. However, whether conclusions drawn from these two techniques in ecological studies are similarly valid has not yet been determined. A systematic comparison of the utility of both approaches is still lacking, and thus, there is a necessity to cross-validate these methods.

In our study, we investigated whether the two methods (i.e., high-throughput sequencing and visual macroscopic assessment of leaves) to study fungal communities would reveal similar patterns of tree diversity effects on foliar fungi. Silver birch (*Betula pendula* Roth), an important broadleaved tree species for forestry in northern and eastern Europe, served as our model system^34^. Our study utilized the long-term Satakunta forest diversity experiment in SW Finland where silver birch has been planted in monoculture, two-, three- and five-species mixtures^35^,^36^. We hypothesized that the molecular approach allows detection of more fungal taxa than the visual macroscopic assessment, and that the fungi detected by visual inspection are a subset of those identified by molecular tools. We tested the hypothesis that there is a positive linear relationship among the most common and abundant fungal taxa from the molecular approach and the macroscopically detectable fungal species. In addition, we hypothesized that fungal community composition, diversity and species distribution patterns are affected by changes in tree species richness.

## Results

### Fungal community of birch leaves as characterized by high-throughput sequencing

High-throughput sequencing of the fungal ITS2 region from birch leaves resulted in 29,849 high quality sequence reads (‘*reads*’), clustered into 184 OTUs and 132 singletons (Supplementary Table S1). Clusters containing birch sequences (14 OTUs) and algae (9 OTUs) were removed, after which 161 OTUs remained that constituted fungal taxa. A core dataset, where each OTU had at least 10 reads, comprised 45 OTUs and 6712 reads in the 55 sampled trees (Supplementary Table S2). Each sample had between 18 and 412 reads, and between 6 and 30 OTUs (median: 103 reads and 18 OTUs per sample). The most common OTUs, which occurred in at least half of the samples, and of which there were 13, accounted for 83% of the reads and 29% of the OTUs.

The fungal community in birch leaves characterized by sequencing had three times more ascomycetes than basidiomycetes (i.e., 34 Ascomycota OTUs, 10 Basidiomycota OTU, and 1 unidentified fungus). Ascomycetes had almost eight times more reads than basidiomycetes (i.e., 5712 reads for Ascomycota, 743 reads for Basidiomycota, and 257 reads for the unidentified fungus). The most common and abundant OTU (i.e., OTU_3) had 1324 reads, and was present in all samples. Given the sequenced ITS2 region, OTU_3 was identified as either *Venturia ditricha* or *Fusicladium peltigericola* with the same level of accuracy. Five other frequently occurring fungal taxa were detected as well, but those OTUs could not be identified to species level (Table 1).

### Fungal community of birch leaves as characterized by visual macroscopic assessment

Visual inspection of the leaves detected *V. ditricha, Discula betulina, Atopospora betulina*, and an unidentified Ascomycota taxon. *V. ditricha* was found on all but one leaf examined, and *D. betulina* was found on 68% of the 547 leaves examined. Both fungal species were detected from all 55 sampled trees, and as such, from all plots and every tree species richness level. *V. ditricha* infestation covered on average 2% of the leaf surface per tree (range: 0.12–9.3%), while *D. betulina* covered 23% of the leaf surface per tree (range: 5–46.5%). However, *A. betulina* and the unidentified Ascomycota species were rarely detected on leaves by visual macroscopic assessment, namely, on 4 and 14 leaves out of 547 leaves, respectively. When *A. betulina* and the unidentified Ascomycota species were detected, their infestation covered on average 0.14% and 0.16% of the leaf surface per tree, respectively. *A. betulina* was detected on four trees out of 55 trees: two trees from a two-species mixture plot, one tree from a different two-species mixture plot, and one from a five-species mixture plot. The unidentified Ascomycota species was detected on 10 out of 55 trees: one tree from a two-species mixture plot, five trees from four different three-species mixture plots, and four trees from two different five-species mixture plots.

### Comparison of methods to detect the fungal community

The relationship among the most abundant OTUs detected by high-throughput sequencing and the most common fungal species detected by visual macroscopic assessment was tested with ordinary linear regressions. No OTUs showed a positive relationship with *D. betulina* infestation. Of the six most abundant OTUs, the relative abundance of OTU_7 had a significant negative relationship with the infestation by *D. betulina* (Table 2). Thus, increased abundance of OTU_7 in the leaves corresponded with less visible damage by *D. betulina* seen on the leaves. There was a trend for a positive relationship between the relative abundance of OTU_3 (putatively identified as a *Venturia* sp., where the genus *Fusicladium* is the anamorph of *Venturia*^37^,^38^) and the infestation by *V. ditricha*. In general, however, there was no positive linear relationship between the fungal taxa detected by high-throughput sequencing and visual macroscopic assessment.

### Tree species diversity effects on the fungal communities of birch leaves

**Figure 1:**
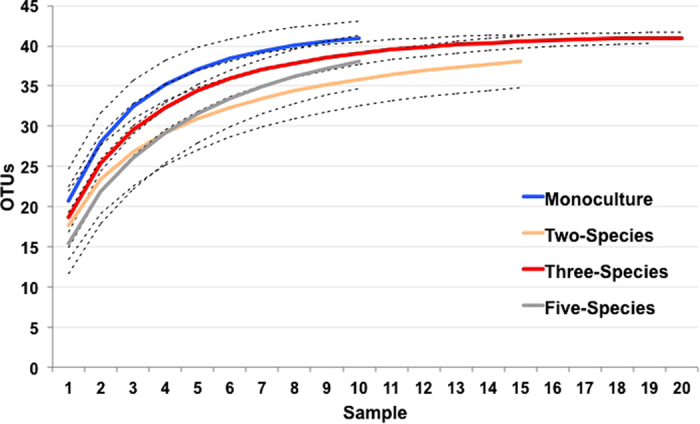
Rarefaction curves present the relationship between number of samples and fungal species richness in operational taxonomic units (OTUs). Each curve represents a different tree species richness level. The blue solid curve represents samples in the monoculture plots, orange solid curve represents samples in the two-species mixture, red solid curve represents samples in the three-species mixture and the grey solid curve represents samples in the five-species mixture. Broken lines represent the 95% confidence interval for each curve.

Fungal OTU richness was similar among the tree species richness levels. From the core dataset, the monoculture plots had 42 OTUs, two-species mixture plots had 39 OTUs, three-species mixture plots had 42 OTUs, and five-species mixture plots had 40 OTUs. There were 35 OTUs out of the 45 OTUs that occurred in all tree species richness levels. Three OTUs were found in all richness levels except the five-species mixture, and two OTUs occurred exclusively in the five-species mixture plots. The remaining five OTUs had mixed distribution across the tree species richness levels, but missing from one (two OTUs) or two (three OTUs) tree species richness levels. Deeper sequencing could reveal more fungal taxa (Fig. 1).

**Figure 2:**
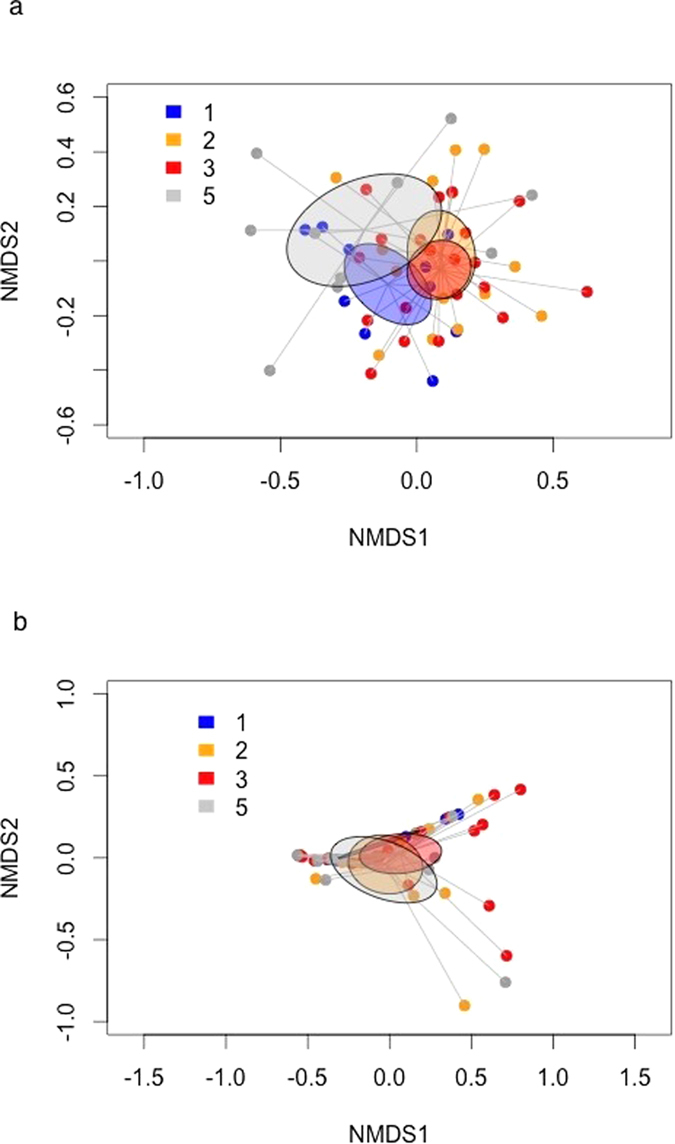
Non-metric multidimensional scaling (NMDS) ordination of (**a**) high-throughput sequence data, based on the core 45 fungal taxa, and (**b**) macroscopic assessment data, based on four fungal taxa. Axes are arbitrary, represent two NMDS dimensions and are scaled in units of Bray-Curtis dissimilarity. A stable solution was reached (stress values = (**a**) 0.18 and (**b**) 0.02). The colored ellipses mark the 95% confidence interval of the group centroids for each tree species richness level. Each tree species richness level is color-coded: monoculture (1, n = 10 samples) is blue, two-species mixture (2, n = 15) is orange, three-species mixture (3, n = 20) is red and five-species (5, n = 10) mixture is grey). Lines link the samples (yellow circles with red border) to the group centroids.

The fungal community composition resulting from high-throughput sequencing was quite distinct among the tree species richness levels, specifically between the monoculture and the two- and three-species mixtures, and the monoculture and the five-species mixture (Fig. 2a), while the community determined by visual macroscopic assessment overlapped considerably (Fig. 2b). Furthermore, the fungal community composition determined by either method was not significantly related to each other (Procrustes correlation coefficient = 0.1392, P = 0.856). Analysis of similarities (ANOSIM) of the fungal communities determined by high-throughput sequencing and visual macroscopic assessment indicated a weak difference among the tree species richness levels (Fig. 2a [sequencing]: ANOSIM statistic R = 0.1602, P = 0.002, Fig. 2b [macroscopic assessment]: R = 0.1604, P = 0.002). Tree species richness accounted for 2.5% of the variation in the sequenced fungal community (PERMANOVA, degrees of freedom (df) = 1, Mean squares = 0.15035, P = 1), and 16.8% of the variation in the fungal community of four taxa from the visual macroscopic assessment (PERMANOVA, df = 1, Mean squares = 0.47975, P = 0.001). Small differences in the sequenced fungal community among tree species richness levels were further supported by Fisher’s alpha diversity indices that did not differ significantly among the tree species richness levels (Fisher’s alpha [tree species richness levels 1, 2, 3 and 5, respectively] = 8.00, 7.26, 7.03 and 8.62, respectively; χ^2^ = 0.21, df = 3, P = 0.9767).

While the fungal communities in general were different among the tree species richness levels, specific fungal taxa may be differentially influenced, not only by tree species mixtures at the plot scale, but also by the microenvironment created by the local tree neighbourhood. Therefore, specific fungal taxa were analyzed for tree species diversity effects and neighbourhood diversity effects. Among the sequenced fungal taxa, correlation between the distribution of the relative abundance of each OTU per sample and tree species richness (Tree Richness) or diversity in the local tree neighbourhood (Neighbourhood; i.e., the proportion of birch in the neighbourhood of the focal tree), respectively, was tested (Table 3). Among these fungal taxa, OTU_7 (putatively identified as Dothideales sp.) showed a negative correlation with tree species richness (Estimate = −0.220, P = 0.03), and a positive correlation with an increased proportion of birch in the local neighbourhood (i.e., negatively correlated with tree species diversity in the neighbourhood) (Estimate = 0.007, P = 3.08E-05) (Table 3). The distribution of the relative abundance of other OTUs was not correlated with either tree species richness or neighbourhood diversity, though OTU_10 (putatively identified as Helotiales sp.) followed similar trends as OTU_7. Among the macroscopically assessed fungal taxa, the infestation by *V. ditricha* on birch leaves correlated negatively with an increased proportion of birch in the neighbourhood (i.e., positively correlated with tree species diversity in the local neighbourhood) (Estimate = −0.021, P = 0.0215) (Table 4). Thus, more birch trees in the neighbourhood of the focal birch tree correlated with decreased *V. ditricha* infestation. The pattern was consistent with the analysis of OTU_3 (putatively identified as *V. ditricha*).

## Discussion

In this study, the effects of tree species diversity on the fungal community of birch leaves were examined by both molecular high-throughput sequencing and visual macroscopic assessment methods to validate that these two methods resulted in the same ecological pattern. High-throughput sequencing of the fungal community in silver birch leaves revealed 11 times greater diversity of fungi than by the macroscopic assessment method, confirming the first hypothesis. One fungal species detected by visual means was also identified by the molecular method. In general, no positive linear relationship between the fungal taxa detected by high-throughput sequencing and macroscopic assessment was found, thereby rejecting the second hypothesis. Fungal community composition, but not fungal diversity or richness, was affected by the presence of other tree species admixed with birch, which partially supported the third hypothesis. Thus, tree species diversity effects on the fungal community can be studied with either method, though the sensitivity and efficiency of fungal taxa detection may vary.

In our study, a weak signal of tree species diversity on the fungal community composition was detected by both methods. In contrast, Nguyen, *et al*. found a clear tree species diversity effect on the fungal community in Norway spruce in a temperate forest^26^. In another study that visually assessed pathogen damages on leaves, no tree species diversity effect on the incidence pathogen damage on silver birch leaves was observed in the mature forest site in northeast Finland^11^. A majority of birch leaves (98%) on every tree in every tree species richness level examined had pathogen damage^11^. The pattern differences across these three studies may reflect site-specific differences, the tree species, and/or age of the trees studied. Despite the limited effect of tree species diversity on the whole fungal community in birch leaves in the current study, tree species richness effects were detected for one fungal taxon, OTU_7 (putatively identified as Dothideales sp.), which decreased in relative abundance with increasing tree species richness, or decreasing proportion of birch in the local neighbourhood. Such a pattern would be expected from density-dependent transmission of pathogens and is consistent with findings of decreasing pathogen load with increasing tree species richness^10^.

While it may be difficult to make inferences about the ecology of the OTU_7, i.e., whether it was a generalist or specialist fungus, as a result of the shallow taxonomic identification achieved, knowing that OTU_7 was likely a Dothideales sp. gives some insights. The order containes saprotrophs, parasitic species, and important plant pathogens^39^. The closest GenBank match for OTU_7 was to an *Aureobasidium* species, a genus that includes pathogens^40^. However, the negative relationship between the relative abundance of OTU_7 and *Discula betulina* infestation may potentially suggest that OTU_7 was an endophyte or a latent pathogen that protected the leaf from *D. betulina* infestation. OTU_7 also showed a significant negative relationship to species richness. Thus, OTU_7 might have decreased in relative abundance because of dilution effects by tree diversity, and as a consequence, in the absence of OTU_7, *D. betulina* could develop better, resulting in the unexpected positive, albeit not significant, relationship of *D. betulina* to tree species richness.

In contrast to OTU_7, *Venturia ditricha*, a dominant foliar endophyte of birch, particularly in Finland^41^,^42^,^43^, was detected by visual macroscopic assessment. *V. ditricha* was found to correlate negatively with the proportion of birch in the local neighbourhood, or correlated positively with increased local tree species diversity. This was surprising considering that the increased proportion of birch in the local neighbourhood should promote *V. ditricha* infestation. More birch in the vicinity would lead to higher birch litter input, where the fungus presumably overwinters, and consequently, increased fungal spore load and infection the following growing season. However, the presence of other tree species may have altered the biotic and abiotic environmental conditions that could interfere with spore transmission or germination of the fungus^44^. Furthermore, the managed condition of the experimental forest, in which the surrounding vegetation and/or microclimate in the canopy may be altered, can result in reduced *V. ditricha* infestation, as previously shown^45^.

In this study, the two methods employed to study the foliar fungal community on birch leaves resulted in detection of the most common fungal species *V. ditrichia* (teleomorph of *Fusicladium betulae*). The anamorphic genus *Fusicladium sensu lato* is a recognized anamorph of the telomorphic genus *Venturia*^37^,^38^. High-throughput sequencing of the fungal barcoding region ITS2 showed that the sequence of *V. ditrichia* had 100% similarity to the sequence of *Fusicladium peltigericola*, and to distinguish these two species molecularly was not possible based on this barcode alone. Whereas *V. ditricha* is a birch endophyte, *F. peltigericola* was first described as a slow-growing isolate from the lichen *Peltigera rufescens*^46^. These two species could be distinguished if the entire ITS region (ITS1 and ITS2) were to be sequenced from the leaf samples. There is 99% sequence similarity over the entire ITS region between the two species^46^. However, to target the entire length of the ITS region would skew the fungal community against those fungal taxa with long sequences instead^47^. On the other hand, if pure cultures of each fungus could be obtained, other genes that may be used to differentiate the two species. However, high-throughput sequencing of these non-standard fungal barcoding genes would not be feasible in complex environmental samples such as leaves^29^. Reference sequence databases are generally not available for these genes to differentiate fungal taxa.

*Discula betulina*, a common foliar pathogen on birch causing characteristic leaf spots, was detected in all leaves in the visual macroscopic assessment method. From our entire sequence dataset, including both the core and satellite dataset, only two sequence reads in one sample partially aligned (95% query cover) with *Ophiognomonia intermedia* (syn *D. betulina, Gnomonia intermedia*)^48^,^49^. In general, one should be weary of the low frequency sequence reads (i.e., 2 reads out of 7134 reads (161 OTUs) in total). Their rarity may not be biologically meaningful and could be sequencing or clustering algorithm artifacts^50^. Regardless, we expected a correspondence between the two methods in the detection of *D. betulina*. One possible reason for not detecting this common species in the sequencing effort, despite its presence in all sampled trees and 68% of all leaves, and with higher infestation than *V. ditricha*, may be technical of nature. The fruiting structures of *D. betulina* on the leaves may be resistant to the milling process and thus its DNA may not have been extracted. Based on the available reference sequences for *D. betulina*, the primers used in this study should have been able to amplify any *D. betulina* DNA that would be present. As no OTUs showed a significantly positive relationship to *D. betulina*, it further supports the interpretation that the DNA of this fungus was not extracted.

*Atopospora betulina* commonly develops stromata on the upper surface of living leaves^51^. The fungus was detected on leaves by visual macroscopic assessment, but not by high-throughput sequencing. Searching the GenBank sequence database revealed that no reference sequence was available for *A. betulina*. The lack of available reference sequences is a major limitation for the molecular identification of fungal species that may be overcome by sequencing previously described fungal culture collections. These collections exist for few fungal species. Furthermore, many more fungal species cannot be isolated and cultivated or have not been described, such as those fungi from tropical and subtropical plant species^52^. Therefore, the majority of fungi would remain underrepresented in sequence databases. Despite these limitations, ecological questions may still be addressed without a taxonomic identification on a particular sequence^53^.

Molecular high-throughput sequencing and visual macroscopic assessment were found to be complementary and useful tools to determine the relationship between foliar fungal communities and tree species diversity. The visual detection method associated the identification of the fungi with their ecological function and phenotype. However, we found that the power of high-throughput sequencing was the detection of many more fungal taxa that would otherwise be missed due to the lack of fruiting structures or symptoms. Where total community sequencing and visual detection both would reveal limited information about the function of the taxa present, obtaining pure cultures of the fungal species would allow for further experiments, though not so easily achieved. However, recent advances in comparative genomics and transcriptomics may help further to characterize the activities of fungi from environmental samples^54^,^55^. In this way, the interaction between host tree species and fungal species can be studied to better determine the potential mechanisms underlying the effects of tree species diversity.

## Material and Methods

### Study area and sampling

To study the effects of tree species diversity on the foliar fungal community of silver birch, sampling was conducted on August 12–15, 2011 at the Satakunta forest diversity experiment (www.sataforestdiversty.org), which is part of the global network of forest diversity experiments TreeDivNetwork^36^, and is located in southwest Finland (61.42°N, 21.58°E) at an elevation of 35 m. The plantation was established in 1999 with one-year-old seedlings in a clear-cut boreal forest to study the effects of tree species richness and composition on various aspects of ecosystem functioning^35^,^36^. The soil was an acidic podzol composed of granodiorite bedrock beneath a clay-textured mineral horizon. In 2011, the mean annual precipitation and temperature was 700 mm and 6.4 °C, as noted from a nearby meteorological station 20 km from the sampling site in Pori^56^.

The study was conducted in one of three Satakunta experiment areas planted with monocultures, two-, three- and five-species mixtures of silver birch, black alder (*Alnus glutinosa* (L.) Gaertner), Siberian larch (*Larix sibirica* Ledeb), Norway spruce (*Picea abies* (L.) H. Karst), and Scots pine (*Pinus sylvestris* (L.)). Limiting the study to one experimental area reduced potential confounding differences in fungal composition between different geographic locations. Individual plots within each area were 20 m x 20 m with trees planted in a grid of 13 × 13 trees at 1.5 m planting distance between and within rows with 169 trees per plot. All tree species in a plot were planted in equal proportion. In 2010, mean tree height was 5–6 m and canopy closure was reached in most plots, except for some monocultures of *A. glutinosa*. In the current study, a subset of 11 birch-containing plots were used: two birch monoculture plots, three two-species mixture (birch-pine, birch-spruce, and birch-alder), four three-species mixture (birch-pine-spruce, birch-pine-larch, birch-pine-alder, and birch-larch-alder), and two five-species mixture plots with all tree species. No additional monoculture and five-species mixture beyond the two included were present in the experimental area. The sampling design took into account the whole diversity gradient and allowed exploration of any potential nonlinearities in tree diversity effects. The mean tree height of birch was 7.4 m.

In each plot, five randomly selected birch trees were sampled, resulting in a total of 55 trees. Trees on the edge of the plot were not selected to avoid edge effects. Fully expanded leaves were sampled from four branches, two from the top and two from the lower third of the canopy; one branch in each canopy layer was north-facing and the other branch was south-facing. Five leaves from each branch, in total 20 leaves per tree, were collected into paper bags and dried at 60 **°**C within 8 to 20 hours after sampling, for three days. Once dried, 10 out of 20 leaves were randomly selected and processed for high-throughput 454 pyrosequencing. The remaining 10 leaves were used for visual macroscopic assessment of fungal species. All of the leaves together from one tree were considered as one sample.

### Sample preparation for high-throughput sequencing

Leaves were not surface sterilized prior to DNA extraction. A sub-sample of 10 leaves per tree was pulverized in a ball mill (Retsch, Haan, Germany), and approximately 50 mg of leaf powder was transferred into a 2 mL screw-cap centrifuge tube. DNA extraction was conducted with 1 mL CTAB buffer (3% cetyltrimethyl-ammonium bromide (CTAB), 2 mM EDTA, 150 mM Tris–HCl, 2.6 M NaCl, pH 8). Protein contamination was removed with 1 part chloroform, DNA was precipitated with 1.5 part 2-propanol, and the DNA pellet was washed with 0.5 mL 70% ethanol. Molecular grade water was used to resuspend the DNA pellet.

The nuclear ribosomal internal transcribed spacer (ITS) region is used as the universal barcode for fungi^29^, consisting of ITS1 and ITS2, separated by the conserved 5.8 S gene. Due to potential biases from sequencing the entire ITS region^53^, the ITS2 region was amplified with the fungal primer fITS7^47^ and primer ITS4^57^ that contained a unique 8-base pair (bp) sample identification barcode for each sample, resulting in amplicons 207-392-bp in length (mean amplicon length, 305 bp). PCR amplification of each sample occurred in 50 μL reactions and contained 0.025 U μL^−1^ DreamTaq DNA polymerase and buffer (Thermo Fisher Scientific, Waltham, MA, USA), 200 μM of dNTPs, 500 nM fITS7, 300 nM ITS4, 2.75 mM MgCl_2_, and 0.125 ng μL^−1^ genomic template DNA (or 1:10 of the extraction control), and performed using an Applied Biosystems 2720 Thermal Cycler (Applied Biosystems, Carlsbad, CA, USA). PCR negative controls (no samples added) were used to evaluate that there was no contamination during the preparation for PCR. The PCR cycle parameters consisted of an initial denaturation at 95 °C for 5 min, 26–35 cycles of denaturation at 95 °C for 30 s, annealing at 56 °C for 30 s and extension at 72 °C for 30 s, followed by a final elongation step at 72 °C for 7 min. The number of amplification cycles was determined individually for each sample to preserve the fungal genotype composition. Thus, the PCR was interrupted while in the exponential phase to yield weak to medium-strong amplicons as visualized by gel electrophoresis on 1% agarose gels as recommended by Lindahl, *et al*.^53^. Amplicons were purified with the Agencourt AMPure XP kit (Beckman Coulter, Brea, USA) and the concentration of each sample was determined using the Qubit Fluorometer 2.0 and dsDNA High Sensitivity assay kit (ThermoFisher Scientific, Waltham, USA). Each sample was amplified in triplicate and each replicate was individually purified and concentration determined. PCR amplicons were mixed in equal concentration of into one general sample, and the pooled sample was further purified with the E.Z.N.A. Cycle-Pure Kit (Omega Bio-tek, Norcross, USA). The pooled sample was subjected to 454 pyrosequencing after ligation of sequencing adaptors. Construction of the sequencing library and high-throughput sequencing with the GS FLX Junior (Roche, Switzerland), which is equivalent to an eighth of a plate with Titanium series chemistry, were conducted by Eurofins MWG GmbH (Ebersberg, Germany). One DNA extraction control was sequenced, while PCR negative controls were not sequenced since nothing was amplified from them. No sequencing reads were obtained for the DNA extraction control.

### Analysis of visual macroscopic assessment

Fungal species on leaves were identified to species level by examining the upper and lower leaf surface using a light microscope, and classifying the fungal structures or damage symptoms according to reference guidebooks or manuals^58^,^59^. The infestation (i.e., total damaged area) of each fungal taxon was surveyed on the upper and lower leaf surface using stereomicroscopy, and was estimated by rating on a scale with seven damage classes: 0%, 1–5%, 6–10%, 11–25%, 26–50%, 51–75% and 76–100%^9^. Fungal infestation per tree individual was calculated by averaging foliar fungal infestation of all analysed leaves of the individual.

### Bioinformatics and data analysis

The sequence reads (“reads”) data generated from 454 sequencing were subjected to quality control and single-linkage clustering in the SCATA bioinformatics pipeline (http://scata.mykopat.slu.se). The SCATA pipeline workflow utilized in data handling has been extensively detailed in Clemmensen, *et al*.^60^. Briefly, high quality sequence reads were obtained by following quality filtering that included choosing the high-quality region extraction (HQR) option (HQR is the longest part of a read that fulfills all the quality thresholds), removal of short sequences (<200 bp), sequences with low mean read quality score (<20), or with score below 10 at any position. Homopolymers were collapsed to 3 bp before clustering, and restored to their original length before final analyses and downstream sequence identification. Sequence reads that were missing the primer sequences or barcode sequence were also excluded. Primer sequences and barcodes were removed, but information on the sequence association with the sample was retained as metadata. Sequences passing quality control were then clustered into different operational taxonomic unit (OTUs) using single-linkage clustering based on 1.5% dissimilarity (i.e. 98.5% similarity) using the USEARCH clustering engine. This clustering threshold approximately corresponds to species level, and was validated by including reference sequences. The most abundant genotype for clusters was used to represent each OTU. For clusters containing two sequences, a consensus sequence was produced. Raw molecular data are stored at the European Nucleotide Archive (ENA) under the accession number PRJEB16069 (www.ebi.ac.uk/ena).

Analysis of the sequence data was conducted on the core dataset, which was defined as those OTUs with 10 or more sequence reads across all samples. Neighbor Joining tree analysis aided in the removal of non-fungal OTUs^53^. BLAST searches allowed the putative identification of OTUs at different taxonomic ranks. Putative species-level assignment was made based on best matches over the entire length of the query sequence and 98–100% sequence similarity, 94–97% sequence similarity for genus level, and 80–93% sequence similarity for order level between, after Ottosson, *et al*.^61^ and Nguyen, *et al*.^26^. Similarity less than 80% has been assigned to class level. Rarefaction curves were constructed separately for each tree species richness level using sample-based data in the software EstimateS version 9.1.0. (R. K. Colwell, http://purl.oclc.org/estimates). The relative abundance of each OTU per sample was determined from the sequence reads as the number of reads for a particular OTU divided by the total number of reads for the sample. *Fungal community composition* was visualized with non-metric multidimensional scaling (NMDS) using Bray-Curtis dissimilarity, with three dimensions specified and 100 random starts. The solution from the first analysis was used as the starting point for a second analysis. Correlation between the ordinations was tested using the Procrustes correlation analysis, where the significance of the congruence between any two ordinations was tested with 999 permutations. The distribution of fungal communities may be a result of tree species richness effects at the plot scale, or more locally at the neighbourhood scale leading to neighbourhood effects. Thus, the proportion of birch was determined from the eight trees immediately surrounding the focal tree. Analysis of similarities (ANOSIM) statistically tests whether there was a significant difference between tree species richness levels, permuted 999 times. Permutational multivariate analysis of variance (PERMANOVA) was used to partition the variance among the community attributed to tree species richness or neighbourhood effects, after constraining by plot, to account for pseudoreplication errors. Significance was computed with 999 permutations. *Fungal community diversity* in each tree species richness level was evaluated with Fisher’s alpha diversity index, which is appropriate for log-normally distributed data such as 454 pyrosequence data. A Chi-Square test evaluated the significant difference between the diversity indices of each tree species richness level. All analyses were conducted in R v3.1.3^62^ using the *vegan* package^63^.

General ecological conclusions may be made about fungal taxa that are abundant, frequent and well represented in each sample. A fungal taxon was considered well represented when the OTU had at least 5 reads per sample, or 275 reads per OTU (5 reads × 55 samples) across all samples. Ordinary linear regressions were used to determine the relationship between the abundant fungal taxa (OTUs) determined by high-throughput sequencing and the fungal species determined by visual macroscopic assessment. To determine the significance of the distribution of individual fungal taxa (OTUs) along the tree species diversity gradient, generalized linear mixed models (GLMMs) for sequence data were used. Effects of plot scale tree species richness and tree neighbourhood on these abundant fungal taxa, respectively, were tested in R using the glmer function in the *lme4* package^64^. Specifically, we tested which, if any, of the most abundant OTUs correlated with tree species richness, and diversity in the neighbourhood of the target tree in terms of the concentration of birch in the neighbourhood. GLMMs take into account the non-independence among sampling units and the hierarchical design of the study^65^. The response variable, which was proportion of a specific OTU in a sample, had a binomial distribution. The link function was a logit function (logit = ln(response probability of OTU/probability of the non-response of OTU)). Odds ratios for the effects of explanatory variable (e.g. tree species richness and the diversity in the neighbourhood of the target tree) can be calculated from the estimated regression coefficients as exponent of the estimated coefficient. In logistic regression, odds ratios are interpreted as a change in the probability of an OTU occurring in the species mixture plots relative to the its occurrence in monoculture plots^66^. Similarly, to determine the significance of the fungal species infestation determined by visual macroscopic assessment along the tree species diversity gradient, linear mixed effect models (LMM, lmer function in R) were used. For both GLMM and LMM, the explanatory variable tested was either tree species richness (Tree richness) or diversity in the local neighbourhood (Neighbourhood); both were continuous fixed factors. Tree richness and Neighbourhood were individually tested in GLMM and LMM. Random factors included plot for both GLMM and LMM, and to properly account for the variance (that does not vary freely), random residual was estimated by specifying the sampling unit, which was a unique identifier for each tree sample for the GLMM.

## Additional Information

**How to cite this article**: Nguyen, D. *et al*. Foliar fungi of *Betula pendula:* impact of tree species mixtures and assessment methods. *Sci. Rep.* **7**, 41801; doi: 10.1038/srep41801 (2017).

**Publisher's note:** Springer Nature remains neutral with regard to jurisdictional claims in published maps and institutional affiliations.

## Supplementary Material

Supplementary Information

## Figures and Tables

**Table 1:** List of the most abundant fungal taxa identified from sequence data.

OTU	Putative taxonomic affiliation	Closest GenBank match	Accession number of closest match	% similarity	Frequency	Number of reads
OTU_3	*Venturia ditricha*	*Venturia ditricha*	KF793774.1	100	55	1324
	*Fusicladium peltigericola*	*Fusicladium peltigericola*	HQ599579.2	100	55	1324
OTU_5	Dothideomycetes sp.			75	55	878
OTU_6	Hypocreales sp.			91	54	680
OTU_1	Helotiales sp.			89	51	570
OTU_10	Helotiales sp.			81	52	503
OTU_7	Dothideales sp.			83	48	385

Putative taxonomic affiliations have been assigned based on percent sequence similarity, with species level between 98–100% similarity, genus level between 94–97% similarity and order level between 80–93% similarity. For sequence similarity less than 80%, putative affiliation has been assigned to class level. The closest GenBank matches and the respective accession numbers are reported. Frequency is the presence of the operational taxonomic unit (OTU) in each sample (n = 55). Number of reads is the sum of the number of reads for the OTU in all samples. All fungal taxa are Ascomycota, and the putative taxonomic ranks of class and order are noted where species-level assignment could not be achieved.

**Table 2:** Simple linear regression model results of the relationship between fungal taxa (i.e., operational taxonomic units, OTUs) detected by high-throughput sequencing and fungal species detected by visual macroscopic assessment.

Fungal species	OTU	Estimate	StdError	P value
*Discula betulina*				
	OTU_3	5.85	13.90	0.6756
	OTU_5	21.75	18.27	0.2392
	OTU_6	16.05	23.18	0.4916
	OTU_1	−15.54	22.81	0.4987
	OTU_10	−31.80	23.00	0.1726
	**OTU_7**	−106.26	26.16	**0.0002**
*Venturia ditricha*				
	OTU_3	7.10	2.80	0.0142
	OTU_5	0.77	3.94	0.8461
	OTU_6	−0.19	4.96	0.9689
	OTU_1	−1.04	4.88	0.8316
	OTU_10	2.89	4.97	0.5631
	OTU_7	−6.87	6.31	0.2811

Relative abundance of each OTU was compared to the percent of fungal infestation for leaves from all silver birch tree samples (n = 55). Significant results are indicated in bold, following Bonferroni correction.

**Table 3:** Generalized linear mixed effect model results of specific fungal taxa (i.e., operational taxonomic units, OTUs) detected by high-throughput sequencing.

OTU	Putative taxonomic affiliation	Explanatory variable	Estimate	StdError	z value	P value
OTU_3						
	*Venturia ditricha* or	Tree Richness	0.003	0.075	0.04	0.97
	*Fusicladium peltigericola*	Neighbourhood	−0.003	0.003	−1.20	0.23
OTU_5						
	Dothideomycetes sp.	Tree Richness	−0.052	0.087	−0.60	0.55
		Neighbourhood	−0.002	0.003	−0.76	0.45
OTU_6						
	Hypocreales sp.	Tree Richness	0.006	0.071	0.09	0.93
		Neighbourhood	0.001	0.003	0.46	0.64
OTU_1						
	Helotiales sp.	Tree Richness	0.070	0.087	0.81	0.42
		Neighbourhood	−0.005	0.003	−1.61	0.11
OTU_10						
	Helotiales sp.	Tree Richness	−0.051	0.091	−0.56	0.58
		Neighbourhood	0.006	0.003	1.81	0.07
OTU_7						
	Dothideales sp.	Tree Richness	−0.220	0.103	−2.13	0.03
		**Neighbourhood**	0.007	0.002	4.20	**3.08E-05**

Effect of tree species richness (Tree Richness) and neighbourhood diversity (i.e., proportion of birch in neighboring trees, Neighbourhood) of the focal tree on the relative abundance of specific fungal taxa in silver birch for all trees (n = 55). Significant results are indicated in bold, following Bonferroni correction.

**Table 4:** Linear mixed effect model results of specific fungal species detected by visual macroscopic assessment.

Fungal species	Explanatory variable	Estimate	StdError	t value	P value
*Discula betulina*					
	Tree Richness	2.750	1.694	1.62	0.10
	Neighbourhood	−0.015	0.057	−0.26	0.80
*Venturia ditricha*					
	Tree Richness	0.325	0.241	1.35	0.18
	Neighbourhood	−0.021	0.009	−2.30	0.02
*Atopospora betulina*					
	Tree Richness	−0.003	0.006	−0.47	0.64
	Neighbourhood	1.10E-04	2.39E-04	0.46	0.64
Ascomycota sp.					
	Tree Richness	0.016	0.009	1.71	0.09
	Neighbourhood	−0.001	3.77E-04	−1.92	0.05

Effect of tree species richness (Tree Richness) and neighbourhood diversity (i.e., proportion of birch in neighboring trees, Neighbourhood) of the focal tree on the relative infestation by specific fungal species on silver birch for all trees (n = 55).
