# Supplementary material for: Foliar fungi of Betula pendula: impact of tree species mixtures and assessment methods
Source: Sci Rep. 2017 Feb 2;7:41801. doi: 10.1038/srep41801 (PMC5288799; doi:10.1038/srep41801)
Supplement: Supplementary Information [file srep41801-s1.pdf]

Supplementary Information

Foliar fungi of silver birch in pure and mixed tree species stands: comparing high-throughput sequencing and morphological assessment

Diem Nguyen*[1], Johanna Boberg[1], Michelle Cleary[2], Helge Bruelheide[3,4], Lydia Hönig[3,4], Julia Koricheva[5], Jan Stenlid[1]

Supplementary Table 1: Fasta formatted sequence data from 454 sequencing with GS FLX Junior that resulted in 184 clusters (i.e., Operational taxonomic units, OTUs) that were of high sequence quality, as enumerated in the Material and Methods. These OTUs have 2 more reads and exclude global singletons.

Supplementary Table 2: Putative taxonomic affiliation of core dataset with 45 fungal OTUs. Fungal OTUs with 10 or more reads and their putative taxonomic assignments and accession numbers based on BLAST searches, number of reads, proportion of reads, and frequency (number of samples where each OTU was detected), represented by each OTU. Putative species level taxonomic affiliation is defined as 98-100% sequence similiarity, genus 94-97%, and order 80-93%.

Supplementary Table 1: Fasta formatted sequence data from 454 sequencing with GS FLX Junior that resulted in 184 clusters (i.e., Operational taxonomic units, OTUs) that were of high sequence quality, as enumerated in the Material and Methods. These OTUs have 2 more reads and exclude global singletons.

>OTU_0
TTAAATTCAGCGGGTAGTCCCGCCTGACCTGGGGTCGCGTTGGAAGCGTC
GCTAGCGCGACACAGCAGGGTCAAGGAGCACACGATGAGCGACGCGGCA
CGCACGACGGGACACGAGGGTTTGACAACCACCGATTGTCGTGGCGCGCG
TCGCCGAGGACTCGCTTTTAGGCCAACCGCATGCATGAGCTCACGGGAGG
CCAATTTCTGCCCCACAGGCCCCCTCGTCCCTTTGCAAGGAGATGGGGTTG
GGGGCAACGATGCGTGACACCCAGGCAGACGTGCCCTCGGCCAGGTGGCT
TCGGGCGCAACTTGCGTT
>OTU_3
TTAAGTTCAGCGGGTATCCCCACCTGATCCGAGGCCAACCTTGTAAAAAT
GGGGGTTCGGGGCGACCGACCGACAGGCACTCCGAGCGAGGGATTTCTCT
TGCTACGCTCAGGGCCGGACACGACGGCGCCCACGGGTTTCGGGCCCGTC
CGCGAGGACGGGGCCCATCACAGAGCAGGGCTCCAGGGTAGAAATGGCG
CTCGAACAGGTGTGCCCCCCGGAATACCAGGGGGCGCAATGTGCGTT
>OTU_5
TTAAGTTCAGCGGGTATTCCTACCTGATCCGAGGTCAACCTTTGAAAAAGT
GAAGGGGAATTCCCGCTCGCGCGGGGTGTTCCGATTCGGTTTCAAGCGGC
AGGAGACTCGAGAGACCCAAGGACGTTTTAGATAAATTCTGCTACGCCCA
AAGCCCAAGAGGCCCCGCCGAAGTGTTTGAGGCCCGCCTCCCGCCGGTGA
GGGCAGGGGAGGCGAAGAGACCTAATACCAAGCGGTGCTTGATTGGTGT
AATGACGCTCGAACAGGCATGCCCCCCGGAATACCAAGGGGCGCAATGT
GCGTT
>OTU_6
TTAAGTTCAGCGGGTATCCCTACCTGATCCGAGGTCAACCTGTAAAAATT
GGGGGTTGTTGGCTAGCCACCAGCAAGACTCAATCGCGAAAAGTATTACT
ACGCGTAGAGCTTACCAGCACCGCCACTAGTTTTAAGGGCCCCGAAGCTG
GGAACCCCAATACCAAGCAAAGGCTTGAGTGGTTATAATGACGCTCGAAC
AGGCATGCCCTTCGGAATACCAAAGGGCGCAATGTGCGTT
>OTU_1
TTAAGTTCAGCGGGTATCCCTACCTGATCCGAGGTCAACCATAGAAAATT
GAGGGTTGCTGGCAAGTTCCCCAGGGTACTCCAAAGCGAGAGAAATTACT
ACGCTTGAAGCCCAGAGGAGCCGCCACTGAATTTGAGGAAAGCCCCGGA
GGGCTGACCCAATACCAAGCAGAGCTTGAGGGTTGTAATGACGCTCGAAC
AGGCATGCCCCCCGGAATACCAGAGGGCGCAATGTGCGTT
>OTU_10
TTAAGTTCAGCGGGTATCCCTACCTGATCCGAGGTCAATACTTAGAAGTTT
GGGGTTCTGGAAGCAAGCGACCACGAGACCTCTGAAAGCGGAATATTTAC
TACGCTTGAGGCTCGACGCCACCGCCAAAGTATTTAAGGCCCGCCCCCGT
TACAGGGACGAAAGCCTAAGGCCAAGCTGTGCTTGATAGTCGTTATATGA
CGCTCGAACAGGCATGCCCTCCGGAATACCAGAGGGCGCAATGTGCGTT

>OTU_7
TTAAGTTCAGCGGGTATCCCTACCTGATCCGAGGTCAACCTGAAAAAGTT
TGGTGTTTTGCGGCAGAAAGGCCCCGCCGGACTCGTGGACGTTATTGAAG
TAATTCTACTACGCCCAAAGCCGATGAGACTTCGCCGAAGGTTTCGGGGC
ACGCCCTCCCCGGTGAGGGGGAGGACGAGGGCCCAATACCAAGCTGTGCT
TGATTGGTGTAATGACGCTCGAACAGGCATGCCCCTCGGAATACCAAGGG
GCGCAATGTGCGTT
>OTU_4
TTAAGTTCAGCGGGTATCCCTACCTGATCCGAGGTCAACCTGAAAAAATT
GGGGGTTTAGCGGCAGAAGGCCTCGCCGGACTCGTGAACGTTTTGAAGTA
ATTCTACTACGCCCAAAGCCGATGAGACTTCGCCGAAGGTTTCGGGGCAC
GCCCTCCCGGGAAGGGGGGACGAAGGCCCAATACCAAGCCAGGCTTGAT
TGGTGTAATGACGCTCGAACAGGCATGCCCCTCGGAATACCAAGGGGCGC
AATGTGCGTT
>OTU_2
TTAAGTTCAGCGCGTATTCCTACCTGATCTGAGGTCAATAGATGAGTGTAG
CCAGGAGCACGAGGCACGAGGCGGGCTTTGGGTGCAGCAACCAAGATTG
CAACCGGCTTGGACAACGAGTTGGACGGACCTAGAACGTTGTGTAACCGG
GCGGCGGGCCGCACCAATGCATTTGAGGAGAGCGTCCTCCCTTGCGAGAG
GTTACGCAGATCCCAACACCCACAGATCACCCAAACAAAGGGTTTCTCTG
GGAGAGATTAATGACACTCAAACAGGCATGCCCTCCGGAATACCAGAGG
GCGCAATGTGCGTT
>OTU_12
TTAAGTTCAGCGGGTAGTCCTACCTGATTTGAGGCCAGATGTCAAGAAGT
TGTAGCCTCTATAAGTGCAGCACTTTGAGACGAGATCAAGCAGTCAAACG
GCCACCCTTGCGAGTGTCCTCAGCGAAATAATTTATAACGCCAAGTCAAA
CCGGCCAACAGGCTAGATCACTACAATATGTTTAAGGTGAGCGGGGCTCA
TCGCCGCGCAAACACCCAAGTCCCAGCCGGACCCTCGGACAAAAGCCCAA
AGGTGAGGTGGATGATTTCATGACACTCAAACAGGCATGCTCCTCGGAAT
ACCAAGGAGCGCAATTTGCGTT
>OTU_8
TTAAGTTCAGCGGGTAGTCCCACCTGATTTGAGGTCTAAATGATATATATA
TTAGGGTTCGGAAGCTATCTATCAAAAGTTCAAATGGCTGTAGTGTTTTTA
AGGCACCACACTCCTCAATGAATAATAAATATTACACCAAGTATATCCAT
TTATTTTCTCAATAAAAGTACTTATATATTTAAGGTGAGCCAATAACGGCA
ACACCCAACATCCATTTCAACTTCTTAACTTAATAAAAAATTGGAATGAG
AGGGTTTCATGACACTCAAACAGGTGTACCTTTCGGAATAACCAAAAGGT
GCAAGGTGCGTT
>OTU_11
TTAAGTTCAGCGGGTATCCCTACCTGATCCGAGGTCAACCTTAGGTGATG
GGTTTTAGGGGCGAGCAACCACCGAGGCCTCTAAAGCTAAAGATTTTACT
ACGCTTGAGGCTCGGGGCCACCGCCGAGGTCTTTAGAGCGCGCCCCGCTG
AGGGGACGGACGCCCAATACCAAGCTGGGCTTGATGGTTGATAATGACGC
TCGAACAGGCATGCCCCTCGGAATACCAAGGGGCGCAATGTGCGTT
>OTU_9
TTAAGTTCAGCGGGTATTCCTACCTGATCCGAGGTCAACCTTGAGAAAAT
AGGTGTGAGACCGTGCCGGAAGACCTTTCAACTCCGTGCGTGCCTTGGAA
AAAGCTCGTTGCACCGAGAGTCTACGCGACGCCGTCATTACTTTTGGGAC
CGGTCGGGGGTGAACCCAGACCGAGAGGTCCAACAACAAGCCAGGCTTG

AGAGGTGATAATGACGCTCGAACAGGCATGCCCTTCGGAATACCAAAGG
GCGCAATGTGCGTT
>OTU_17
TTAAGTTCAGCGGGTAGTCCTACCTGATTTGAGGTCAGAGCACAAAATAT
GGGACGTTATGAGCAAGAGTCGGCACAGCACCAGACGAAACGTATTACA
TCGGGCGACGGCGTATCCCACTAAGTCTTTTGAGGCGAGCCAAGGCGGCA
GCGCCCAAATCCAAGCCACCCGGAAACCCGGGGGTTGAGAGTTTCATGAC
ACTCAAACAGGCATGCCTTTCGGAATACCAAAAGGCGCAAGGTGCGTT
>OTU_13
TTAAGTTCAGCGGGTATCCCTACCTGATCCGAGGTCAACCTGAAAAATAA
AAGGTTTAACGGCAATGAGATCTCACCAAGCTTATAAGACGTTTTGATTT
AACTCTACTACGCCCGAAGTTGGAGAAACCCCGCCGAGGTCTTCGAGGCA
CGCCCGCCTTTCGACGGACGATGCCCAATACCAAGCGGTGCTTGAGTGGT
GTAATGACGCTCGAACAGGCATGCCCCTCGGAATACCAAGGGGCGCAATG
TGCGTT
>OTU_18
TTAAGTTCAGCGGGTATCCCTACCTGATCCGAGGTCAACCATTGAAAGAA
TATGGGCTTGTCCGGCCGGTCCTGCCCGGGGCGGCTGAGCGAGAAGGTTT
TAGTTACTACGCTGAGCTCCCCGGCGAATCCGCCGCTAATTTCCGGGCGC
GCCCCGTGGGGCGGGCGCCCAATACCAAGCCAGGCTTGAGGGTTGTAATG
ACGCTCGAACAGGCATACTCCCCGGAATACCAGGGAGTGCAATGTGCGTT
>OTU_14
TTAAGTTCAGCGGGTAGTCCTACCTGATTTGAGGCCAGATGTCAAAATAT
GTTGCCTCGTTAGAAGCGAGACCAGAGTCCATGATCGGCCACCCCGAAGG
ATGTCCTTAGCGAAATAACTTATAACGCCAAGTCAAACCGGCCATTAGCC
ACTAGTCAGACCGAAATTCATTTAAGGTGAGCCAGCTCTCGCAGGCAAAC
ACCCAACTCCCAGCCAGAACCCCGGACAAAAGCCCAAAGTAGAGGTGGA
TGATTTCATGACACTCAAACAGGCATGCTCCTCGGAATACCAAGGAGCGC
AATTTGCGTT
>OTU_16
TTAAGTTCAGCGGGTATCCCTACCTGATCCGAGGTCAACCTTAGGTGATG
GGTTCTAGGGGCAAGCAACCGCCGAGGCCTCTGAAGCTGGAGATTTTACT
ACGCTTGAGGCTCGGGGCCACCGCCGAGTGCTTTAGGGCGCGCCCCCCGT
TACGGGGACGGGCGCCCAATACCAAGCTGGGCTTGATGGTTGATAATGAC
GCTCGAACAGGCATGCCCCTCGGAATACCAAGGGGCGCAATGTGCGTT
>OTU_26
TTAAGTTCAGCGGGTATCCCTACCTGATCCGAGGTCAACCTTAGGTGTTTG
TTTCTCGAGGCCAAACGATGCCGAACCCAGAAAGCTAAAGATTTTACTAC
GCTTGAGGTTCAACACCACCGCCGAATTCTTTAGGGTGCGTCCCGTGAGG
ACGGCGCCCAATGCCAAGCGGTGCTTGATGGTTGATAATGACGCTCGAAC
AGGCATGCTCCCCGGAATACCAGGGAGCGCAATGTGCGTT
>OTU_15
TTAAGTTCAGCGGGTATCCCTACCTGATCCGAGGTCAACCTTAGAAATGG
GGTTGTTTTACGGCGTAGCCTCCCGAACACCCTTTAGCGAATAGTTTCCAC
AACGCTTAGGGGACAGAAGACCCAGCCGGACGATTTGAGGCACGCGGCG
GACCGCGTTGCCCAATACCAAGCGAGGCTTGAGTGGTGAAATGACGCTCG
AACAGGCATGCCCCCCGGAATACCAGGGGGCGCAATGTGCGTT
>OTU_19
TTAAGTTCAGCGGGTATCCCTACCTGATCCGAGGTCAACCTTGAGGTTATT
TGGTGTTTTGGAAGCGGGCAGACGACGGAGTCCTCGGAAAGCGGGATATG

TTACTACTACGCTTGAGGCTCCGCGCCGCCGCCGAGGTCTTTAGGGCCCGT
CCGCAGCAGGGACGAAGACCCAATGCCAAGCAGTGCTTGATGGTCTATAA
TGACGCTCGAACAGGCATGCCCTCCGGAATGGCCGGAAGGCGCAATGTGC
GTT
>OTU_20
TTAAGTTCAGCGGGTATCCCTACCTGATCCGAGGTCAACCTTTAAAAAAT
AGGTGCTGTTGCGCGGCAAAAGTCCCACCGTCCCACAGAGCGTTATTGAT
AAATTCTACTACGCCCAAAGCCGGTGTGGCTCCGCCGAAATGTTTGAGGC
ACGCCCGCCAGTTACGGGGGCGATGGCCTAATACCAAGCGAGGCTTGATT
GGTGTAATGACGCTCGAACAGGCATGCCCCTCGGAATACCAAGGGGCGCA
ATGTGCGTT
>OTU_28
TTAAGTTCAGCGGGTGTTCTTGCCTGACCTCAGGTCGAAAGCCAAAAGGC
GTCCTGCACAACGCATTCGGGAGCACAGCGGGCAACGAAGGCATGCAAC
CGGCAAAGGTCACCTACCAAATCGGAACTCGCCTTTCCTCGAAAGGAAAG
AGGCTCCCAAAGCGTGAATGTATTTCAGCTGACCGGCCACCCAAAGGGCA
AACCGGACGGCCAGATCCAACTGCCTTTCACCGGAATTCACCAATGAAAT
TTAGTTGAGGGTAGTAGCCGACGCTGAGGCAGACATGCTCTTGGCCGAAG
CCTCGAGCGCAATTTGCGTT
>OTU_21
TTAAGTTCAGCGGGTAGTCCTACCTGATTTGAGGCCAAAATAGAAAAATT
ATATCTCGAAAGATATGACTAGAAAGCAGACTTCATATTATGAGAGTTCA
AAATGTTACTTAACAACTCTGCTGAAGTCCAGCTAATTCATTTAAGGTAAG
CTTGTGTTCGTAACAAACCGCAAGCAGACCCAAGGACCTGACTCGTTGCA
TTTTAATTAAAAAATGCAAGAGCAGAGATATTCAAGACTCTCAAACAGGC
ATGCTCCAAGGAATACCAAGGAGCGCAAGGTGCGTT
>OTU_34
TTAAGTTCAGCGGGTATTCCTACCTGATCCGAGGTCAACCTTGAGATGCG
GTTTCCCGCGCTTGCAAGGTCGGCAGGAGACTCGCCGGGCCCAAGGACGT
TTTCAAGAATGATTTCTGCTACGCCCAAAGCCAGTGAGACTTCGCCGAGG
TTTTCGAGGCCCGCCCCCCGAGAGGGGGACGAAGGGGCCCAATGCCAAG
CTGAGCTTGATTGGTGTAATGACGCTCGAACAGGCATGCCCCCCGGAATG
CCAGGGGGCGCAATGTGCGTT
>OTU_27
TTAAGTTCAGCGGGTAGTCCTACTTGATTGGAGAACAAGCAAAAAATTGA
TGAATGCATCTTGTAAGACGCAGTCAATCAGTTTATCGAGCCTACTTCAAT
TGAATATTGCAACACTATTGAGAAAATTACAACAATAGTTAGCCGAAGCC
TGCATGAATATATTTCAAAGTTTCAGCGACTTAATTTTCAACATAGTTCGA
TAACCTAAGTTATCAACAAACGTCAAGATCTAAACCCTGCTACTCATCAA
TTAAGATAAATGGGGTTTAAGCTTTTCCGTTACTCCAACAAGCATGCTTCC
AGGAATGACCCGGAAGCGCAAGATGCGTT
>OTU_22
TTAAGTTCAGCGGGTATCCCTACCTGATCCGAGGTCAAGAGTGTAAAAAT
GTACTTTTGGACGTCGTCGTTGTGAGTGCAAAGCGCGAGATGTACTGCGC
TCCGAAATCAATACGCCGGCTGCCAATTGTTTTAAGGCGAGTCTACACGC
AGAGGCGAGACAAACACCCAACACCAAGCAGAGCTTGAAGGTACAAATG
ACGCTCGAACAGGCATGCCCCATGGAATACCAAGGGGCGCAATGTGCGTT
>OTU_25
TTAAATTCAGCGGGTAGTCTCGCCTGATCTGGGGTCGTGTTGGAAGCGTC
GCTAGCGCGACACTGCAGGGTCAAAAAGCACACGATGAGTGACATGGCA

CGCACGACGGGACACGAGGGTTTGTCAACCACCGATTGTCATGGCGCGCG
TCGCCGAGGACTCGCTTTTAGGCCAACCGCATGCATGAGCTCAAGGGAGG
CCAATTTCCGCCCCACAGGCCCCCCTCGTCCCTTTGCAAGGAGATGGGGTT
GGGGGCAACGATGCGTGACACCCAGGCAGACGTGCCCTCGGCCAGGTGG
CTTTGGGCGCAACTTGCATT
>OTU_24
TTAAGTTCAGCGCGTATTCCTACCTGATCTGAGGTCAATAAATGAGGTGTA
GCTAAGGGCACAGAGGCACATAGCGGGCTTCAGGTGCAGCAACCATTGTT
GCAACCGGCATAATCAACGAGTTGGACGAACCTAGAACGTTGTGTAACCG
GGCGGCGGGCCGCACCAATTCATTTGAGGACAGCGAAGTTCAAAAGAAA
CTCGCAGATCCCAACACCGACAGAGAACCAAATAAGAGTTCTCTGTGAGA
GATTAATGACACTCAAACAGGCATGCCCTCCGGAATACCAGAGGGCGCAA
TGTGCGTT
>OTU_33
TTAAGTTCAGCGGGTATCCCTACCTGATCCGAGGTCAACCTTGAGGTGAG
AGGTTTCTCGAGGCCGGACGGTCCCAAAACCTAGAAAGCTGATAAAAAGT
TTACTACGCTTGAGGTTTGAAACCGCCGCCGAGGCGTTTAAGGCACGTCC
GGTGGGGGACGACACCCAATACCAAGCAGAGCTTGATGGTTGAAAATGA
CGCTCGAACAGGCATGCTCCCCGGAATACCAGGGAGCGCAATGTGCGTT
>OTU_23
TTAAGTTCAGCGGGTAGTCCTACCTGATTTGAGGTCAGAGCAAAAAATGT
CAGAAGACGGGTTATGAGCAGCCCTCCGGTCTTGGCCAGCGAAACTTATT
ACGCCAGATTCGACCTCGGAACCGCTAACACTTTTAAGGCGAGCCAGAGA
CGGCAACACCCAGATCCATCGTCATCGGAAACCCGATGGGATGAGGTTTT
CATGACACTCAAACAGGCATGCCCTTCGGAATACCAAAGGGCGCAAGGTG
CGTT
>OTU_29
TTAAGTTCAGCGGGTATCCCTACCTGATCCGAGGTCAACCTGTAAAAATT
GGGTTTTTAGCGGCAAAGGAGGCCTGCCGGGTCTTGAACGTGGAGAAATT
CTACTACGCCCAAAGCCGGAGAGGCTCCGCCGAGGTGTTTGAGGCACGCC
CACCTTTCGGAGGACGATGGCCTAATACCAAGCAGAGCTTGAGTGGTGTA
ATGACGCTCGAACAGGCATGCTCCCCGGAATACCAGGGAGCGCAATGTGC
GTT
>OTU_37
TTAAGTTCAGCGGGTATTCCTACCTGATCCGAGGTCAACCATTGAAGATG
GGTGCTTGTCCGGCGGATCCGCCCCGGGCCGCTGAGCGTGCAAATGTTAC
TGCGCTGAGTGCCGGGGACGGGATCGCCGCTGACTTTCGGGCGCGCCCCG
GGGGGCGGGCACCCAATACCAAGCCGGGCTTGAGGGTTGTAATGACGCTC
GAACAGGCATACTCTCCGGAATGCCAGAGAGTGCAATGTGCGTT
>OTU_30
TTAAGTTCAGCGGGTATCCCTACCTGATCCGAGGTCAACCTAGAAAATTT
AAAGGTTTAACGGCAATGAGATCCGACCAAGCTTATAAGACGTTCGATTT
AACTCTACTACGCCCGAAGTTGGTTGAACCCCGCCGAGGTCTTCGAGGAA
GGCCCGCCTTTCGACGGACCGTTCCCAATACCAAGCGATGCTTGAGTGGT
GTAATGACGCTCGAACAGGCATGCCCCTCGGAATACCAAGGGGCGCAATG
TGCGTT
>OTU_47
TTAAGTTCAGCGGGTATTCCTACCTGATCCGAGGTCAACCTTGTTAAAATA
AAAGGAGACCGGGCCCGAGAACCTTTCAACTCTGATACGTGCTGGTGAAA
GCTCATTGTACCGAGGGTCCCACGGACGCCGTCATTGTCTTTGGAACCGGT

CGCGCCACGAGGGCGGGACCAAAAGTCCAACAACAAGCCAGGGCTTGAG
AGGTGATAATGACGCTCGAACAGGCATGCCCTTCGGAATACCAAAGGGCG
CAATGTGCGTT
>OTU_36
TTAAGTTCAGCGGGTATCCCTACCTGATCCGAGGTCAACCTGAGATGGGG
TGTTTAGCGGCGTCGCGCCCTCCGTTCTCCTAGCGGGATTGTGTAATCAAA
TCGCTTCGAGGACGTTAGGCTGAGCCGAAGACATTGGGGCGCGCGGCGGA
CCGCGGCGCCCAATACCAAGCGAGGCTTGAGTGGTGAAATGACGCTCGAA
CAGGCATGCCCCTCGGAATACCAAGGGGCGCAATGTGCGTT
>OTU_39
TTAAGTTCAGCGGGTATCCCTACCTGATCCGAGGTCAAAAGTTGAAAAAA
AGGCTTAATGGATGCTAGACCTTTGCTGATAGAGAGTGCGACTTGTGCTG
CGCTCCGAAACCAGTAGGCCGGCTGCCAATTACTTTAAGGCGAGTCTCCA
GCAAAGCTAGAGACAAGACGCCCAACACCAAGCAAAGCTTGAGGGTACA
AATGACGCTCGAACAGGCATGCCCTTTGGAATACCAAAGGGCGCAATGTG
CGTT
>OTU_31
TTAAGTTCAGCGGGTGTTCTTGCCTGACCTCAGGTCGAAAGCCAAAAGGC
GTCCTGCACAACGCAATTGAGATCATAGCGGGCAACGAAGGCATGCAACC
GGCAAAGGTCACCTACCAAATCGGAACTCGCCTTTCCTCGAAAGGAAAGC
AGTTCTCAAAGCGTGAATGTATTTCAGCTGACCGGCCACCCGAAAGGCGA
ACCGGACGGCCAAATCCAACTGCCTTTCATCGGAATTCACCAATGAAGCT
TAGTTGAGGGTAGTAGCCGACGCTGAGGCAGACATGCTCTTGGCCGAGGC
CTCGAGCGCAATTTGCGTT
>OTU_32
TTAAATTCAGCGGGTAGTCCCGCCTGACCTGGGGTCGCGTTGGAAGCGTC
GCTAGCGCGACACAGCAGGGTCAAGGAGCACACGATGAGCGACGCGGCA
CGCACGACGGGACACGAGGGTTTGACAACCACCGATTGTCGTGGCGCGCG
TCGCCGAGGACTCGCTTTTAGGCCAACCGCATGCATGAGCTCACGGGAGG
CCAATTTCTGCCCCACAGGCCCCCTCGTCCCTTTGCAAAGAGATGGGGTTG
GGGGCAACGATGCGTGACACCCAGGCAGACGTGCCCTCGGCCAGGTGGCT
TCGGGCGCAACTTGCGTT
>OTU_41
TTAAGTTCAGCGGGTATCCCTACCTGATCCGAGGTCAACCTGGTATAAAA
TGATTTTTTTGACGGCGGAAGACTCCCCGGGCCTGTAGGACGTTTTGAAA
GATTCTACTACGCCCAAAGCCGAGGTGACTTCGCCGAAGGTTTTGAGGCA
CGCCCACCCTCTCGGGGGGCGAAGGCCTAATACCAAGCGGTGCTTGATTG
GTGTAATGACGCTCGAACAGGCATGCCCCCCGGAATACCAAGGGGCGCA
ATGTGCGTT
>OTU_42
TTAAGTTCAGCGGGTATCCCTACCTGATCCGAGGTCAACCTAAGAGGTGT
TGGTCATTGGAGGCGGGCGAACCGCAGGCCACACGAAGCGGGATGTATG
TTACTACGCTTCGGGGACTGGGCCACCGCCGAGTTCTTTAGGGCGCGTCCC
GGTGAGGGGGCGGCGCCCAAGGCCAAGCAGAGCTTGATGGGTTGTAATG
ACGCTCGAACAGGCATGCCCCTCGGAATACCAAGGGGCGCAATGTGCGTT
>OTU_48
TTAAGTTCAGCGGGTATTCCTACCTGATCCGAGGTCAACCTTGTAAAAAAT
TTAGGTAAACGACCGGGTCCATGAGACCGCTCGGTGCGTGCTCGTGAAAG
CTCGTTGCACCAAGGGCCTCATAGGCACCGTCATTATCTTTGGGACCGGCC
CCGTTAAGGGCCAGAGGTCCAACACCAAGCCAGGCTTGAGGGGTGATAAT

GACGCTCGAACAGGCATGCCCCCCGGAATACCAAGGGGCGCAATGTGCGT
T
>OTU_50
TTAAGTTCAGCGGGTGTTCTTGCCTGACCTCAGGTCGAAAGCCAAAAGGC
GTCCTGCACACGCTTTCATGAAAGCCTTGGCGAGCAACGAAGGCATGCAA
CCGGCAAAGGTCACCTACCAAATCGGAACTCGGCTTTCCTTTTTAGGGGA
AAGCAGCTTCCACAGCATGAATATTTTTCAGCTAACCGGCAACCTAGTCG
GAACACCGGAAAGCCAGCTCCAATCAATTACCCATACGGGAATTGGTTGA
GGGTAGTAAGCCGACGCTGAGGCAGACATGCTCTTGGCCGAGGCCTCGAG
CGCAATTTGCGTT
>OTU_35
TTAAGTTCAGCGGGCATTCCTACCTGATCCGAGGTCAACCTTAAGAAGTT
GGGGTTGCTGGTCGCCAACTGCCAGGACCCGTAGCGAGGTGAGTACTACG
CTCGGGACCCAACGGTGCCGCCACTAAATTTAAGGCCTGCCATCACTGGC
ACGGCCTAATACCAAGCAGAGCTTGAATGGTGTATATGACGCTCGGACAG
GCATGCCCCTCGGAATACCAAGGGGCGCAATGTGCGTT
>OTU_40
TTAAATTCAGCGGGTAGTCCCGCCTGACCTGGGGTCGCGTTGGAAGCGTC
GCTAGCGCGACACAACAGGGTTAAGGAGCACACGATGAGCGACGCGGCA
CGCACGACGGGACACGAGGGTTTGACAACCACCGATTGTCGTGGCGCGCG
TCGCCGAGGACTCGCTTTTAGGCCAACCGCATGCATGAGCTCACGGGAGG
CCAATTTCTGCCCCACAGGCCCCCTCGTCCCTTTGCAAGGAGATGGGGTTG
GGGGCAACGATGTGTGACACCCAGGCAGACGTGCCCTCGGCCAGGTGGCT
TCGGGCGCAACTTGCGTT
>OTU_57
TTAAGTTCAGCGGATAATCCTGCCTGATTTGAGATCTAATCAAAAGGTAG
ACTTTTAAATTAGGAGCTTCCTTAATGAAGTTGGCTCGATGTTTCCACCGA
ATCCTTAGCGAATAGTCTATTATGCCAAGTCAATCCAAACTTGTATTAGGG
ATGCTAATGTATTACGAACGAGCTAGGCCGAAGCCAGCAGCGCTCAAAAT
CCAAACACCAATCGATTACAAGAAAAGATTGGGTTGAAGAATTCATGACA
CTCAAACAGACATGCTCTCCGGAATACCAGAGAGCGCAAGATGCGTT
>OTU_43
TTAAGTTCAGCGGGTATTCCTACCTGATCCGAGGTCAACCAGTGAGAAGG
GTTCCCATCGGCGGGGGCCGAAGGGCTCCGTTGGACGTCGCGATGTGCTC
TACCACGCCCGAAGCCCGCTCGACTCCGCCGAGGCATTTGAGGCCGGCCC
GCCCGGCGAGGGGCGGACGGCGGCCTAATACCAAGCGGTGCTTGATGGTT
TTAATGACGCTCGAACAGGCATGCCCCCCGGAATGCCAAGGGGCGCAATG
TGCGTT
>OTU_44
TTAAGTTCAGCGGGTAGTCCTACCTGATTTGAGGTCAAATTTGATGATGAT
TTTGTCCGAAGACCATTATAAGCGGACTCTGAGGGATCAAACGACAGCGC
AACATGATTATCACACTGACATCCGGAAGCCAAAGGTTGAATTCCGCTGA
TGCATTTGAGAGGAGCCGACCAATATGCGGCCAGCACCTCCAAGTCCAAT
CTCCGAAAGGTCGTGAAACCCTTGGAGTTGAGAATTTAATGACACTCAAA
CAGGCATGCTCCTCGGAATACCAAGGAGCGCAAGGTGCGTT
>OTU_46
TTAAGTTCAGCGGGTAGTCCTACCTGATTTGAGGCCAAAATAGAAAATTG
TCTATAAAGACGATTGGAAAGCAGACTTCATATTACGAGAGTTCAAAATG
TTACTTAACAACTCTGCTGAAGTCCAGCTAATTCATTTAAGGTAAGCTTGT
GTTCGTAACAAACCGCAAGCAGACCCAAGGACCTGACTCGTTGCACTTTA

GCTAAAAAATGCAAGAGCAGAGATATTCAAGACTCTCAAACAGGCATGCT
CCAAGGAATACCAAGGAGCGCAAGGTGCGTT
>OTU_45
TTAAGTTCAGCGGGTAGTCCTACCTGATTTGAGGCCAAAATAGAAAATGT
TCTCTCGAAAGAGAATGATTAGAGAGCAGACTTCATATTACGAGAGTTTT
CAATGTTACTTAACAACTCTGCTGAAGTCCAGCTAATTCATTTAAGGTAAG
CTTGTGTTCGTAACAAACCGCAAGCAGACCCAAGGACCTGACTTGTTACC
TTTTCGTTAAAAAAAGGCAAAAGCAGAGATATTCAAGACTCTCAAACAGG
CATGCTCCAAGGAATACCAAGGAGCGCAAGGTGCGTT
>OTU_68
TTAAGTTCAGCGGGTATCCCTACCCGATCCGAGGTCAACCTTGAAGGTGTT
TGGTGTGCTGGGGGCGAGCGACGCCGCGCCTCAGGAAGCGAGAAGATTCT
ACTACGCTTAGAGGCTCGGGGCCACCGCCGAGATGTTTGGGGCGCGTCCT
CTTCCGGAAGGAAAGGGCGATACGTCCAATGCCAAGCGGTGCTTGATGGT
TTGTAATGACGCTCGAACGGGCATGCCCCCCGGAATACCAGAGGGCGCAA
TGTGCGTT
>OTU_58
TTAAGTTCAGCGGGTATCCCTACCTGATCCGAGGTCAACCTTAAAAGAAT
GGGGGTTATGGCAAGTGTTCTCCCGGGGACTATAGCGAGAAGAATTACTA
CGCTTAGAGCCGAGAGGCACCGCCACTGATTTTAAGAGCTGGGAGATCCC
AAGCCCCAAGACCCCCGAAGGGATTGGTATTAATGACGCTCGAACAGGCA
TGCCCCCCGGAATACCAAGGGGCGCAATGTGCGTT
>OTU_59
TTAAGTTCAGCGGGTATTCCTACCTGATCCGAGGTCAACCTTGAGGTGCG
GTCTCCCGCGCTTAGAGGTCGGCAGGAGACTCGCCGGGCCCCAGGACGTT
TTCAAGAATGATTTCTGCTACGTCCAAAGCCGGTGAGACTTCGCCGAGGT
TTTTGAGGCCCGCCCCCCGGGAGGGGGACGAAGAGGCCCAATGCCAAGCT
GAGCTTGATTGGTGTAATGACGCTCGAACAGGCATGCCCCCCGGAATGCC
AGGGGGCGCAATGTGCGTT
>OTU_49
TTAAGTTCAGCGGGTAGTCCTACTTGATTTGAGTTTAATAATCACTTTGTG
TGCAAAAGACACGTTTGTGGTAAGCTCTTTCAACTTTAATGCGTTCCTATT
GAATTTAAGTTACAACAATAGGTCAAGCCGCAAGTGTGTTGATTCAGCTT
GTTGCATTTCAACGAAGTCGGCTGTTACATCGACAAACGTCATGATCCAA
AGCCCAAACAACTCTCGATAAACGAAAGCGGGGTTGAGAGGTTTTAAAA
GCTCAAACAAGCATGCTCCCAGGAATAACCCGAGAGCGCAAGGTGCGTT
>OTU_52
TTAAGTTCAGCGGGTATCCCTACCTGATCCGAGGTCAACCTTTGAAAGATT
TAACGGCCATGCCGAACCGAACTCCACTGCGAAATAGTTGCCACAACGCT
GGGAGACGGGTCGTACAGCCGGAGACTTTGAGGCGCGCGAGAGACTCGC
GACGCCCAATACCAAGCGAGGCTTGAGTGGTGAAATGACGCTCGAACAG
GCATGCCCCCCGGAATACCAGGGGGCGCAATGTGCGTT
>OTU_63
TTAAGTTCAGCGGGTATTCCTACCTGATCCGAGGTCAACCTTGTAAAAATT
TAGGTAAACGACCGGGTCTATGAGACCGCCAAGTGCGTGCTCGTGAAAGC
TCGTTGCACCAAGGGCCTCATAGGCACCGTCATTATCTTTGGGACCGGCCC
TGTTTAGGGGCCAGAGGTCCAACACCAAGCCACGCTTGAGGGGTGATAAT
GACGCTCGAACAGGCATGCCCCCCGGAATACCAAGGGGCGCAATGTGCGT
T
>OTU_97

TTAAGTTCAGCGGGTAGTCCTACCTGATTTGAGGCCAAAATTGAAAAATT
ATTAGATAGTCAACAAAGACTAGTCTGATTAGAAAGCAGATTTCCTATTA
CGAGAGTTTTCAATGTTACTTAACAACTCTACTGAAATCCAGCTAATTCAT
TTAAGGTAAGCTTGTGTTCGTAACAAACCGCAAGCAGACCCAAGGACCTG
ACTCGTTTTATCTCTCGTTAAAAAAATAAAGAGCAGAGATATTCAAGACA
CTCAAACAGGCATGCTCCAAGGAATACCAAGGAGCGCAAGGTGCGTT
>OTU_38
TTAAGTTCAGCGGGTAGTCCTACCTGATTTGAGGTCACATTAATGAAAAA
TTTGTCCAAGTCAATGGACTGTTAGAAGCTGAACCTGCCTCACGTCAATA
GCGTAGATAGTTATCACACCAATGAACGGTCAACAAATTGTCCCGCTAAT
ATATTTTAGGGGAGCTGACCTCAAAGAGAGACCAGCAAAAAACTACCCCC
ACATCCAACACTCGACAAACAAAGCTGAAGAGGTTGATATGTTAATGACA
CTCAAACAGGCATGCTCCTCGGAATACCAAGGAGCGCAAGGTGCGTT
>OTU_55
TTAAGTTCAGCGGGTATCCCTACCTGATCCGAGGTCAAAGTAAAAAAGGT
CTTGTGGAAGAGGGCCTGTTGAATTCGCTTGCAATGTGCTGCGCACGAAG
CCAACATACCGGCTGCCAATGAATTTGAGGCGAGTCCACGCGCTGAGGCG
GAACAAACACCCAACACCAAGCATAGCTTGAAGGTTTAAATGACGCTCGA
ACAGGCATGCCCAACGGAATACCGAAGGGCGCAATGTGCGTT
>OTU_56
TTAAGTTCAGCGGGTAGTCCTACCTGATTTGAGGCCAAAGATATAATGAA
TAAAATTCCGTTAGAAAGCAGACTTCATATTACGAGAGTTTTCAATGTTAC
TTAACAACTCTACTGAAATCCAGCTAATGCATTTAAGGCAAGCGTGTGTTC
GTAACAAACCGCAGGCAAGCCCAAGGACCTGACTCCAAACCAACTTTCGT
TAAAAAATTGAGGAGCAGAGATATTCAAGACACTCAAACAGGCATGCTCC
AAGGAATACCAAGGAGCGCAAGGTGCGTT
>OTU_98
TTAAGTTCAGCGGGTATTCCTACCTGATCCGAGGTCAACCTTAGAAAAATT
TGTGGTTAAGACCAAGCCGGACAGAAACAAACTGCGATACGTGCCTTGTG
CAAGACTCATTGTACCGAGGCTTCCGTCAAGCCTGTCATTATCTTTGGGAC
CCGGGCCCGGAGGCCCGTAGAAAAAGTCCAACACCAAGCCAAGCTTGAG
GGGTGATAATGGCGCTCGAACAGGCATGCCCTTCGGAATGCCAAAGGGCG
CAATGTGCGTT
>OTU_51
TTAAGTTCAGCGGGTAGTCCTACCTGATTTGGGGCCAGATCAATGAAAGT
AGTTGTCCGAAGACGGGTTGGAAAGCAGACGACGTCCAGGGGTGGCCTCC
GACGTGTCCTCAGCGAAATACTTATTACGCCAAGTCAAACCATCCCAAGG
GCTAGTCCAGCTAATGCATTTGAGGCGAGCCGACCGCGAGGCCGGCAAAC
GCCCAAGTCCAGCCGTGCCCGAGGAGTTAACCAAAGGCAAGGTTGATTGT
TTTCATGACACTCAAACAGGCATGCCCGGCGGAATACCACCGGGCGCAAG
GTGCGTT
>OTU_53
TTAAGTTCAGCGGGTAGTCTCACCCGATTTGAGGTCAAGGTTTTCGTTTTG
TGGGTCCAGCCTCCGCCAATGTAGATGGCGGTGACCAGAGCACCATCATT
GTCCGTGTGGGACAGTTAGGAGCAGGCGTCCAAGGGACAGTGCTAGATCC
AAAACGTAGAAGCATCTTATCACGTTAAGGATCAGCAAAGTGGACCCCAC
TAATTCATTTGAGAGGAGCTCACTTTCAAAAGTGAAAGGCAAGCATTGAA
CCTCCAAGTCCAAAATCCCATTCCTGACAGGAAAAGGGTTTTGAGGATTT
CACGACACTCAAACGGGTGTGCCCCTCGGAATGCCAAGGGGCGCAAGGT
GCGTT

>OTU_60
TTAAGTTCAGCGGGTATCCCTACCTGATCCGAGGTCAACCTTGATAAAATT
GGGGGTTACTGGCAAGACACCACCGAGACCCTATAGCGAGAAGAATTACT
ACGCTCAGAGCTCGAGGGCACCGCCACTGGGTTTAGAGGCTGCGGATCG
CAGGCCCCAAGACCAAGCCGAAGCTTGATGGGTTGAAATGACGCTCGAAC
AGGCATGCCCCGCGGAATACCACGGGGCGCAATGTGCGTT
>OTU_78
TTAAGTTCAGCGGGTATTCCTACCTGATCCGAGGTCAACCTTTGAAAATTG
AGAATTGCTTCTCGCTAAGGCCGGGCCACAAAACCTTTCGATGTATGCTGT
ATAAGCTCGCTGCATCTGGGGTCATTTGTGACGCCGCCATTATCTTTCGGG
CCTATCTTTAACGATAAGAGACCCAATAACAAGCCGGGCTTGAGGGTTGA
TAATGACGCTCGAACAGGCATGCCCTTCGGAATACCAAAGGGCGCAATGT
GCGTT
>OTU_99
TTAAGTTCAGCGGGTATCCCTACCTGATCCGAGGTCAACCTAGAAAATAA
AGGTTTCAGTCGGCAGAGTTCCTCTCCTTTGACAGACGTTCGAATAAATTC
TACTACGCCTAAAGCCGGAGTGGCCTCGCCGAGGTCTTTAAGGCGCGCCC
AACTAAGGACGACGCCCAATACCAAGCATAGCTTGAGTGGTGTAATGACG
CTCGAACAGGCATGCCCCTCGGAATACCAAGGGGCGCAATGTGCGTT
>OTU_147
TTAAGTTCAGCGGGTATCCCTACCTGATCCGAGGTCAACCTGAAAAAAAT
GGAGGTTTTGACGGCGGAAGACGCACCGGGCCCACAGGACGTTTTGAAA
AAATCTACTACGCCCAAAGCCGGAGTGACTTCGCCGAAGGTTTTGAGGCA
CGCCCACCCTCTCGGGGGACGAAGGCCTAATACCAAGCGGTGCTTGATTG
GTGTAATGACGCTCGAACAGGCATGCCCCCCGGAATACCAAGGGGCGCA
ATGTGCGTT
>OTU_61
TTAAGTTCAGCGGGTAGTCCTACCTGATTTGAGGTCCAGCATAAAGGATC
TGCCCAAAGGCATGGGGTTCTGAGCAGGCATGTGCCATCCAAGGTCAGAC
GAAACTTATCACGTCAGGCCAGAGGAAACACTTCCCACTAACGTCTTTAA
GGCCAGCCGGTTGCCCGGCAGGGCCCATCTCCAAGTCCACCAGACCACAG
TCGCAAAACCGGGGGGGATTGATGTTTATAAGACACTCAAACAGGCATGC
CCCTCGGAATACCAAGGGGCGCAAGGTGCGTT
>OTU_62
TTAAGTTCAGCGGGTAGTCCTACTTGATTGGAGAACAAGCAAAAAATTGA
TGAATGCATCTGTAAGACGCAGTCAATCGGTTTATCGAGCCTACTACAATT
GAAAATTTGCAACACTATTGAGAAAATTACAACAATAGTTAGCCGAAGCC
TGCATGAATATATTTCAAAGTTTCAGCGACTTGATTTTCAACATAGTTGAT
AACCTAAGTTATCAACAAACGTCAAGATCTAAACCCTGCTACTCATCAAT
TAAGATAAATGGGGTTTAAGCTTTTCCGTTACTCCAACAAGCATGCTTCCA
GGAATGACCCGGAAGCGCAAGATGCGTT
>OTU_73
TTAAGTTCAGCGGGTAGTCCTACCTGATCCGAGGTCAAAGTTGAAAAAAA
GGTGGGGGTTGTCAGCGGGCGGGCGCGCCCGGCCAGACGAAACGTATCA
CGTCGGGCACGGTCGCTCCTCTCACTAAGGCATTTCAGGCGAGCCGAGGT
GGCAACGCCCAGGTCCAAGTCCGCCCGGGGGAAACCCCGGGTGGATTGAT
GTTTTCATGACACTCGAACAGGCATGCCTTTCGGAATACCAAAAGGCGCA
AGGTGCGTT
>OTU_75

TTAAGTTCAGCGGGTATCCCTACCTGATCCGAGGTCAACCTTTTGAGGTAT
GGTTTTTCTGGAAGCTAGCGGTCGCGAGGCCCCCGAAGCGGGATATTTCT
ACTACGCTTGAGGCTCCGCGCCGCCGCCGAGGTCTTTGGGGCCCGTCCGC
TGCGGGGACGAAGGCCCAATGCCAAGCTGTGCTTGAGGTTCTTAAATGAC
GCTCGAACAGGCATGCCCCCCGGAATACCAAGGGGCGCAATGTGCGTT
>OTU_80
TTAAGTTCAGCGGGTATTCCTACCTGATCCGAGGTCAACCTTTGAAAATAA
AAGATCGACGGCGGAGTCCACCGGTGCTCTCGACGTCAGAAATTCTAACA
CGCCGGGGGTCGGTGTCCTCGCCGATGCATTTCGGGCGGGCCGGGCGCCG
GAGCGCGGGCCAACGCCTAATACCAAGCGGGGCTTGATTGGTGAAATGAC
GCTCGAACAGGCATGCCCCCCGGAATACCAGGGGGCGCAATGTGCGTT
>OTU_84
TTAAGTTCAGCGGGTAGTCCTATCTGATTTGAGGTCTAAAGCTTAAATGAA
TAATGCATTTGTAGCACCACTAACGTTTTCAAAGCCTGTCAAAACGTCCTC
AGCGAAATAGTTATTACGCCAAGTCAAACCGTTCAAATGGTACATTGAAA
AGTGGGTCGCTCGTATATTTCAGTCGAGCCGGTCTCTGACGAGTCCGACA
AACAACCATAATCCAAGCCCACAGCCTGTCTCATTACAGAACAGGGGGGT
TGAGAGTTTCATGACACTCAAACAGACATACTCTTCGGAATACCAAAGAG
TGCAAGGTGCGTT
>OTU_92
TTAAGTTCAGCGGGTATTCCTACCTGATCCGAGGTCAACGTTCAGAAGTTG
GGTGTTTTACGGCGTGGCCACGTCGGGGTTCCGGTGCGAGTTGGATTACT
ACGCAGAGGTCGCCGCGGACGGGCCGCCACTTCATTTCGGGGCCGGCGGT
ATGCTGCCGTTCCCCAACGCCGATTTCCCCAAAGGGAAGTCGAGGGTTGA
AATGACGCTCGAACAGGCATGCCCGCCAGAATGCTGGCGGGCGCAATGTG
CGTT
>OTU_54
TTAAGTTCAGCGGGTAGTCCTGACTGATTTGAGGTCTAATGTAAAAAAGT
AGTGCTTTGTTAGCCCGCTTAACAAACGTCGACAGACTCTGTTCTTAACGA
AATAATTATCACGCCAAGTCGAAAGTCTGTGCTATTACATTTGATAGCTTC
AGCTCTTGTATTTAGACCGAGTCGGCAATGAAGCGACATTAGACGATCAA
CATCCAAAGCCCGAGACTCTTTCGTCATTACAACAAAAGGGGGTTTTGAG
AGATTCACGACACTCAAACAGACATACTCCTGGGAATACCCAAGAGTGCA
AGGTGCGTT
>OTU_66
TTAAGTTCAGCGGGTATCCCTACCTGATCCGAGGTCAACCTTTGTTAAAGA
GGTTTTTACGGCGCGGGTCCCCTAACTCGAGAGCGAAACGATGCTTCTAC
GCTCGGAGGTCGGGTGCTCGGCCGGTGTCATTGAGGCTCGCGTGTCCGCG
GAGCCCAAAACCAAGCTGTGCTTGAGTGGTGTAATGACGCTCGAACAGGC
ATGCCCCCCGGAATACCAGGGGGCGCAATGTGCGTT
>OTU_69
TTAAGTTCAGCGGGTATCCCTACCTGATCCGAGGTCAACCTGGTGTGGTGT
GTTTGGACGGCGTCAAACCCTCCGATCCCGCAAGCGATTTGGGTTGCCAC
AACGCTTCGAGACGGACGGCTCAGCCGGAGACATTGGGGCGCGCGGCAG
GCCGCGACGCCCAAGACCCAGCGAGGCTGGACTGGTGTAATGACGCTCGA
ACAGGCATGCCCCCCGGAATACCAGAGGGCGCAATGTGCGTT
>OTU_71
TTAAGTTCAGCGGGTATCCCTACCTGATCCGAGGTCAACCATAGAAAAAT
TTGGGTTTTGGCAGAAGCACACCGAGAACCTGTAACGAGAGATATTACTA
CGTTCAGGACCCAGCGGCGCCGCCACTGATTTAGAGCCTGCCATTACTG

ACATAGACTCAATACCAAGCTAAGCTTGAGGGTTGAAATGACGCTCGAAC
AGGCATGCCCCCCGGAATACCAAGGGGCGCAATGTGCGTT
>OTU_72
TTAAGTTCAGCGGGTATCCCTACCTGATCCGAGGTCAAAGTAAAAAGGAG
CTTAATGGAAGGGGGTCTATCAGAATCGCATCGCAATGTGCTGCGCGCGA
AGCCAACTTACCGGCTGCCAATGAATTTAAGGCGAGTCCACGCGCAGAGG
CGGGACAAACACCCAACACCAAGCAGAGCTTGAAGGTTTAAATGACGCTC
GAACAGGCATGCCCAACGGAATACCGAAGGGCGCAATGTGCGTT
>OTU_76
TTAAGTTCAGCGGGTATCCCTACCTGATCCGAGGTCAACCTGAAAAAATT
TGGGTGTTTTAGCGGCAGAAGGCCCCGCCGGACTCGTGGACGTTATTGAA
GTAATTCTACTACGCCCAAAGCCGATGAGACTTCGCCGAAGGTTTCGGGG
CACGCCCGCCCCGGTGAGGGGGGACGAAGGCCCAATACCAAGCTGTGCTT
GATTGGTGTAATGACGCTCGAACAGGCATGCCCCTCGGAATACCAAGGGG
CGCAATGTGCGTT
>OTU_82
TTAAGTTCAGCGGGTATCCCTACCTGATCCGAGGTCAACCTGTAAAAATA
TTGGGGGTTACTGGCAAGACGCCACCGAGACTCTATAGCGAGGATAATTA
CTACGCTCAGAGCTCAATGGCACCGCCACTGGGTTTAGAGGCTGCGAAAC
CGCAGGCCCCAAGACCAAGCCGAAGCTTGATGGGTTGAAATGACGCTCGA
ACAGGCATGCCCCGCGGAATACCACGGGGCGCAATGTGCGTT
>OTU_89
TTAAATTCAGCGGGTAGTCCCGCCTGACCTGGGGTCGCGTTGGAAGCGTC
GCTAGCGCGACACAGCAGGGTCAAGGAGCACACGATGAGCGACGCGGCA
CGCACGACGGGACACGAGGGTTTGACAACCACCGATTGTCGTGGCGCGCG
TCGCCGAGGACTCGCTTTTAGGCCAACCGCATGCATGAGCTCACGGGAGG
CCAATTTCGGCCCCACGGGCCCCCCTCGTCCCTTTGCAAGGAGATGGGGTT
GGGGGGCAACGATGCGTGACACCCAGGCAGACGTGCCCTCGGCCAGGTG
GCTTCGGGCGCAACTTGCGTT
>OTU_108
TTAAATTCAGCGGGTAGTCCCGCCTGACCTGGGGTCGCGTTGGAAGCGTC
GCTAGCGCGACACAGCAGGGTCAAGGAGCACACGATGAGCGACGCGGCA
CGCAGGACGGGACACGAGGGTTTGACAACCACCGATTGTCGTGGCGCGCG
TCGCCGAGGACTCGCTTTTAGGCCAACCGCATGCATGAGCTCACGGGAGG
CCAATTTCTGCCCCACAGGCCCCCTCGTCCCTTTGCAAGGAGATGGGGTTG
GGGGCAACGATGCGTGACACCCAGGCAGACGTGCCCTCGGCCAGGTGGCT
TCGGGCGCAACTTGCGTT
>OTU_118
TTAAGTTCAGCGGGTATCCCTACCTGATCCGAGGTCAACCTTGAGGTGAG
ATGTTCTCGAGGCAGGACGGCCACAACCTAGAAAGCTAAGGATTTCTACT
ACGCTTGAGGTTTGTAACCGCCGCCAAAGGGTTTAAGGCGCGTCCACGGG
GGACGACGCCCAATGCCAAGCAGTGCTTGATGGTTGATAATGACGCTCGA
ACAGGCATGCTCCCCGGAATACCAGGGAGCGCAATGTGCGTT
>OTU_157
TTAAGTTCAGCGGGTATTCCTACCTGATCCGAGGTCAACCTTAGAAATGG
GGGGTTTTACGGCAAGAACCCGCCGCACGACCATAGCGATGTAGAGTTAC
TACGCTCGGTGTGACTAGCGAGCCCGCCACTGATTTTGAGGGACCGCGGA
CAGCCGCGGATCCCCAACGCAAGCAGAGCTTGATGGTTGAAATGACGCTC
GAACAGGCATGCTCGCCAGAATACTGGCGAGCGCAATGTGCGTT
>OTU_191

TTAAGTTCAGCGGGTATCCCTACCTGATCCGAGGTCAACCTTGAGGTGAT
GGGTTTTGGAAGCGGGCGACAAAAAGACCTCTGAAGCGGGATAAATTTTA
CTACGCTTGAGGCTCTGTGCCACCGCCGAATCCTTTGGAGCGAGCCCCCA
GTTACGGGGGCTGACGCCCAATGCCAAGCAGAGCTTGATGGTTGATAATG
ACGCTCGAACAGGCATGCCCTTCGGAATACCAAAGGGCGCAATGTGCGTT
>OTU_64
TTAAATTCAGCGGGTAGTCCCGCCTGACCTGGGGTCGCGTTGGAAGCGTC
GCTAGCGCGACACAGCAGGGTCAAGGAGCACACGATGAGCGACGCGGCA
CGCACGACGGGACACGAGGGTTTGACAACCACCGATTGTCGTGGCGCGCG
TCGCCGAGGACTCGCTTTTAGGCCAACCGCATGCGTGAGCTCACGGGAGG
CCAATTTCTGCCCCACAGGCCCCCCTCGTCCCTTTGCAAGGAGATGGGGTT
GGGGGCAACGATGCGTGACACCCAGGCAGACGTGCCCTCGACCAGGTGG
CTTCGGGCGCAACTTGCGTT
>OTU_65
TTAAGTTCAGCGGGTATCCCTACCTGATCCGAGGTCAAAGGTTGAAAAAA
AGGCTTGCTGGACGCTGACCTTGGCTGGCAAAGAGCGCGACTTGTGCTGC
GCTCCGAAACCAGTAGGCCGGCTGCCAATGACTTTAAGGCGAGTCTCCAG
CGAACTGGAGACAAAAGACGCCCAACACCAAGCAAAGCTTGAGGGTACA
AATGACGCTCGAACAGGCATGCCCTTTGGAATACCAAAGGGCGCAATGTG
CGTT
>OTU_67
TTAAATTCAGCGGGTAGTCCCGTCTGACCTGGGGTCGCGTTGGAAGCATC
GCTAGCGCGACACAGCAGGGTCAAAGAGCACACGATGAGCGACGCAGCA
CGCACGACGGGACACAAGGGTTTGTCAACCACCGATTGTCGTGGCGCGCG
TCGCCGAGGACTCGCTTTTAGGCCAACTGCATGCATTAGCTCACGGGAGG
CCAATTTCTGCCCCACAGGCCCCCTCGTCCCTTTGCAAGGAGATGGGGTTG
GGGGAAACGATGAGTGACACCCAGGCAGACGTGCCCTCGGTCGAGGTGG
CTTCGGGCGCAACTTGCGTT
>OTU_70
TTAAGTTCAGCGCGTATTCCTACCTGATCTGAGGTCAATAAATGAATAGC
AAGGAGCACAAGGCACCAGGCGGGCTTAAGGTGCAGCACCATGAATGCA
CCGGAGAGCAACGAGTTAGACGAACCTAGAACGTTGTGTAACCGGGCGG
CGGGCCGCACCAATACATTTGAGGAGAGCGAACTCGAGAGGTCGCAAGT
CCCAACTTCGACAGAGACCAGACGGTTTGAAACCAAATGGTCTTGTGAGA
GATTAATGACACTCAAACAGGCATGCCCTCCGGAATACCAGAGGGCGCAA
TGTGCGTT
>OTU_83
TTAAGTTCAGCGGGTGTTCTTGCCTGAGCTCAGGTCGAAAGCCAAAAGGC
GTCCTGCACACGCTTTCATGGAAGCCATTGCGAGCAACGAAGGCATGCAA
CCGGCAAAGGTCACCTACCAAATCGGAACTCGGCTTTCCTTTTTGAAGGG
AAAGCAGCTTCCACAGCGCGAATGTTTTTCAGCTGACCGGCAACCTAGTC
GGAAAACCGGAAAGCCAGCTCCAACCAACTTCCACAAGGGAATTGATTG
AGGGTAGTAAGCCGACGCTGAGCCAGACATGCTCTTGGCCGAAGCCTCAA
GCGCAATTTGCGTT
>OTU_88
TTAAGTTCAGCGGGTATCCCTACCTGATCCGAGGTCAAACTTAGAAGTTG
GGGGTTGCTGGCAAGCACGCACCGAGGACCCTGTAGCGAGGAGTATTACT
ACGCTTAGAGCACGATGGCACCGCCACTGATTTTAAGACCGATCAGAACT
GATGCGGCCCAATACCAAGCAGAGCTTGAGTGTTGTACTGACGCTCGAAC
AGGCATGCCCCGCGGAATACCACGGGGCGCAATGTGCGTT

>OTU_91
TTAAGTTCAGCGGGTATTCCTACCTGATCCGAGGTCAACCTTAGAAAAAA
AGGGGGGTATAAGACCGTGGCTCGCGCACCCTAGATTCGATACGTGCTCA
GTTAAGAAGCTCAGTGTACCGGGGGTCCAACGAGGCCGCCGTCGTTGTCT
TTAGGAGGGGTCCAGGTGACCTGGACCAAACCGTCCAAAACCAAGCCCG
GGGGCTTGAGGGGTGTAAATGACGCTCGAACAGGCATGCCCTTCGGAATA
CCAAAGGGCGCAATGTGCGTT
>OTU_93
TTAAGTTCAGCGGGTAGCCCTACCTGATTTGAGGCCAGATCATGAATATG
TGGGGTTATCAGCCACCCAGAAGGATGAAACGTATTACATCCAAGGTGCT
TATGTCTTTAAGGCGAGCCTTTAGCAAGGCAACACCCAATACACCACCGC
TCAGGCAAAAACCCAAGTGGGGTGAGGTTTCATGACACTCAAACAGGCAT
GCCCTTCGGAATACCAAAGGGCGCAAGGTGCGTT
>OTU_96
TTAAGTTCAGCGGGTACTCCTACCTGATCCGAGGTCAACCTTTAAGTTAAA
AGTTTTGACGGCTGAAGACCCCCCGGCCACACGGACGTTTTAACTAGATT
CTGCTACGCCCGAAGCCGGATTGACTCCGCCGAAGGTTTTGTAGGCCCGC
CCAGCGTTTGACCGCGGACGGAAGGCCTAATGCCAAGCTGTGCTTGATTG
GTGTAATGACGCTCGAACAGGCATGCCCCCCGGAACTACCAAGGGGCGCA
ATGTGCGTT
>OTU_100
TTAAGTTCAGCGGGTATCCCTACCTGATCCGAGGTCAAAGACGGTAAATG
TGCTTGTTGGACGCGAACCGGAGCCCCTCAAGAAGCGCAATGTGCTGCGC
GAGAGGAGGCAAGGACCGCTGCCAATGAATTTGGGGCGAGTCCACGCGC
GGAGGCGGGACAGACGCCCAACACCAAGCAGAGCTTGAGGGTGTAGATG
ACGCTCGAACAGGCATGCCCCACGGAATACCGAGGGGCGCAATGTGCGTT
>OTU_101
TTAAGTTCAGCGGGTATCCCTACCTGATCCGAGGTCAACCTTGAGAGTGC
GCTGGGCGCGGTTTTACGGCTGGGGCCCGCGGAGGTCGGACGTCTGGGGT
CTCCCCCTTTCTGATACGCCCGGCCGACGCGAGGTCCTGCCGACTCTTTTG
GGGCACGCCCTGGCCGGAGCCGGGGGCGAAGCCCATCGCCAAGCGGTGC
TTGATGGTTGTAATGACGCTCGAACAGGCATGCCCCCCGGAATGCCGAGG
GGCGCAATGTGCGTT
>OTU_107
TTAAGTTCAGCGGGTATCCCTACCTGATCCGAGGTCAAATTGAAAATATTC
GCTTCATGGACGCTGGACCACTAGACCAAAGCGCAAAAAATGTGCTGCGC
TCCGAAACCAGTAGGCCGGCTGCCAATGATTTTAAGGCGAGTCTCGGGGC
GGACCCAAGACAAAAACGCCCAACACCAAGCAAAGCTTGAGGGTACAAA
TGACGCTCGAACAGGCATGCCCTTTGGAATACCAAAGGGCGCAATGTGCG
TT
>OTU_112
TTAAGTTCAGCGGGTATCCCTACCTGATCCGAGGTCAACCTGATAGAAGT
GGTTTAACGGCGCGGGCGCCCCTCCTCGACAGCGGATTTGTGTGTCACAA
CGCTGAGATCGGGGTCGTCCAGCCGGCGAAATTGGAGCGCGGGTCGCCCC
CGGCGCTCAAGACCAAGCCAGGCTTGAGTGGTGTAATGACGCTCGAACAG
GCATGCCCCTCGGAATACCAAGGGGCGCAATGTGCGTT
>OTU_129
TTAAGTTCAGCGGGTATCCCTACCTGATCCGAGGTCAACCTTTGATGGAG
GTTTTAACGGCGCGATTCCCCTTGCCCTCGAGCGAAAGTTTTGTGCTTCTG
CGCTCGGAGATCGGGTGACCGGCCGGTGATATTGGGGCGCGCGTTTGACC

GCGGCGCCCAACACCAAGCGAGGCTTGAGTGGTGTAATGACGCTCGAACA
GGCATGCCCTCCGGAATACCAGAGGGCGCAATGTGCGTT
>OTU_139
TTAAGTTCAGCGGGTGTTCTTGCCTGACCTCAGGTCGAAAGCCAAAAGGC
GTCCTGCACACGCTTTCATGAAAGCTTTTGCGAGCAACGAAGGCATGCAA
CCGGCAAAGGTCACCTACCAAATCGGAACTCGGCTTTCCTTTTTAGGGGA
AAGCAGCTTCCACAGCGTGAATATTTTTCAGCTAACCGGCAACCTAGTTG
GGACACCGGAAAGCCAGCTCCAATCAATTACCCATAGGGGAATTGGTTGA
GGGTAGTAAGCCGACGCTGAGGCAGACACGCTCTTGGCCGAAGCCTCGAG
CGCAATTTGCGTT
>OTU_144
TTAAGTTCAGCGGGTATTCCTACCTGATCCGAGGTCAACCTTGGTGGGGTC
GGTCTTTGCAGACCTGGTTTTACGGCCGGAAAGCTCCTCCGCGGGCCTCCT
AAGCGAGATATTTTACTACTGCGCTCGGAGCCGCGGCGGGTCCGCCACTG
TCTCTGGGGGCCTCCCGCGAGGGAGAGCCCCGACGCCAGGCACCAGGGCC
TGAGGGGTTGAAATGACGCTCGAACAGGCATGCCCGCCAGAATGCTGGCG
GGCGCAATGTGCGTT
>OTU_159
TTAAGTTCAGCGGGTATCCCTACCTGATCCGAGGTCAAAAGTTAAAAATG
TAGAGTCTTGATGGATTACCGTCCTTTTCTCCTGATACAAAGCGCAAAATA
TGTGCTGCGCTCCGAAACCAGTAGGCCGGCTGCCAATCGTTTTAAGGCGA
GTCTCCCAGAAAGAGGGAGACAAAAAACGCCCAACACCAAGCAAAGCTT
GAAGGTACAAATGACGCTCGAACAGGCATGCCCTTTGGAATACCAAAGG
GCGCAATGTGCGTT
>OTU_244
TTAAGTTCAGCGGGTATTCCTACCTGATCCGAGGTCAACCTTAGATAAGTG
ACTTTAACGGCATGAACCACCCGTCTCGCCAGCGAATTAGTTTCCACAAC
GCTGAGATAGAGATTGTCCAGCCGGTCGAAATTGAGGCGGGGGACTGGTT
ACCCGTCGCCCAATACCAAGCCAGGCTTGAGTGGTGTAATGACGCTCGAA
CAGGCATGCCCTTCGGAATGCCAAAGGGCGCAATGTGCGTT
>OTU_251
TTAAGTTCAGCGGGTAGTCCTACCTGATTTGAGGTCAGAGGTCATAAAGT
TGTCTCTCAAGTGAGACGGTTAGAAGCTCGCCAAACGCTTCACGGTCGCG
GCGTAGATATTATCACACCGAGAGCCGATCCGCAAGGAATCAAGCTAATG
CATTTAAGAGGAGCCGACCGATCAAGGCCGACAAGCCTCCAAGTCCAAGC
CTACAAACCACAAAAGCTTATAGGTTGAAGATTTCATGACACTCAAACAG
GCATGCTCCTCGGAATACCAAGGAGCGCAAGGTGCGTT
>OTU_300
TTAAGTTCGGCGGGTATCCCTACCTGATCCGAGGTCAACCTTGAGGTGAG
AGGGTTCTCGAGGCAAAAACGGTCCCAAACCTAGAAAGCTGAAAAGATTT
TACTACGCTTGAGGTTTGAAACCGCCGCCGAGGCGTTTAAGGCACGTCCG
TACGGACGACGCCCAATTCCAAGCAGAGCTTGATGGTTGATAATGACGCT
CGAACAGGCATGCTCCCCGGAATACCAGGGAGCGCAATGTGCGTT
>OTU_74
TTAAATTCAGCGGGTAGTCCCGCCTGACCTGGGGTCGCGTTGGAAGCGTC
GCTAGCGCGACACAACAGGGTCAAGGAGCACACGATGAGCGACGCGGCA
CGCACGACGGGACACGAGGGTTTGACAACCACCGATTGTCGTGGCGCGCG
TCGCCGAGGACTCGCTTTTAGGCCAACCGCATGCATGAGCTCACGTGAGG
CCAATTTCTGCCCCACAGGCCCCCCTCGTCCCTTTGCAAGGAGATGGGGTT

GGGGGCAACGATGCGTGACACCAGGCAGACGTGCCCTCGGCCAGGTGGC
TTCGGGCGCAACTTGCGTT
>OTU_77
TTAAATTCAGCGGGTAGTCCCGCCTGACCTGGGGTCGCGTTGGAAGCGTC
GCTAGCGCGACACAGCAGGGTCAAGGAGCACAGTGATGGAGCGACGCGG
CACGCACGACGGGACACGAGGGTTTGACAACCACCGATTGTCGTGGTGCC
GCGTCGCCGAGGACTCGCTTTTAGGCCAACCGCATGCATGAGCTCACGGG
AGGCCAATTTCTGCCCCACAGGCCCCCCTCGTCCCTTTGCAAGGAGATGG
GGTTGGGGGCAACGATGCGTGACACCCAGGCAGACGTGCCCTCGGCCAG
GTGGCGTTCGGGCGCAACTTGCGTT
>OTU_79
TTAAGTTCAGCGGGTATCCCTACCTGATCCGAGGTCAATGGTATAATATGT
TTGGGCTTCTGGAAGCGGGCGACGCCGAGACCCCTGAGAGCGGAAGAATT
CACTACGCTCGAGGCTCGTCGCCACCGCCGACCGATTTAGGGGGCGCCCC
GCGAGGGGCGGGCTCCCAAGGCCAAGCAAGGTGCTTGATTGTCCACAACA
TGACGCTCGAACAGGCATGCCCTCCGGAATACCAGAGGGCGCAATGTGCG
TT
>OTU_81
TTAAGTTCAGCGGGTATCCCTACCTGATCCGAGGTCAACCGTAAGAGTTG
GGGGTTGTTGGCAAGCCACCCTCCGGACCCTGTAGCGAGAAAATTTACTA
CGCTTAGAGCCAGACGGCACCGCCACTGATTTAGGGGCCGCGACGCCGC
GAACCCCAATACCAAGCTAGGCTTGAGTGGTCATAATGACGCTCGAACAG
GCATGCCCCTCGGAATACCAAGGGGCGCAATGTGCGTT
>OTU_85
TTAAGTTCGGCGGGTAGTCTCACCCGATTTGAGGTCAAGGTCTTTTTTGTG
GGTCCAGCCTCCCGCCAATGTAGATGGCAGTGACCGAAGCACCATCGTCG
TCCAACAGACGGTTAGGAGCAGGCGTCCAAGGGACAGTGCCAAATCCAA
AACGTAGAAACATCTTATCACGTCAAGGATCAGCAAAGCAGACCCCACTA
ATTCATTTGAGAAGAGCTCGCTTTCAAAAGCGAGCATAGAACCTCCAAGT
CCAAAAAATCCTTTTCCCGACCAAAAGAGGATCAAAGGAAAAGGTTTTGA
TGATTTCACGACACTCAAACGGGTGTGCCCCTCGGAATGCCGAGGGCGCA
AGGTGCGTT
>OTU_86
TTAAGTTCAGCGGGTATCCCTACCTGATCCGAGGTCAACCTTTGATAAAAT
TGGGGGTTGCTGGCAGGCACGCGCCCGGTCCTCCAGAGCGAGAAGGATTA
CTACGCTTGGAGCCGGACGGCGCCGCCACTGATTTTGAGGCGCACCGGGA
CGGTGACACCCAAGACCAAGCAGAGCTTGAGGGTTGTAATGACGCTCGAA
CAGGCATGCCCCCCCGGAATACCAGAGGGCGCAATGTGCGTT
>OTU_87
TTAAGTTCAGCGGGTAGTCCTACCTGATTTGAGGCCAAAGAAAATGAATA
TGCGTCAATAAAGACGGCTAAAGTTATGAGCAGATTCCGTATTACGAGAG
TTTTCAATGTTACTTAACAACTCTACTGAAATCCAGCTAATACATTTAAGG
CAAGCTTGTCTTTAGTAACAAAGCGCAAGCAAGCCCAAGGACCTGACTCC
AAACCAACTTTAATTAAAAAAATTGAGGAGCAGAGATATTCAAGACACTC
AAACAGGCATGCTCCAAGGAATACCAAGGAGCGCAAGGTGCGTT
>OTU_90
TTAAGTTCAGCGGGTATCCCTACCTGATCCGAGGTCAACCTGAAAAAAAT
TGGGGGTTCTGGCAAGTAACCAGGGGGAACTCCATCGCGAGAAGAATTAC
TACGCGTAGAGCCCACCGGCACCGCCATTGATTTTAGGGGCTGCGAGAC

CGCAAACTCCAATACCAAGCCAGGCTTGAGTGGTTATAATGACGCTCGAA
CAGGCATGCCCTGCGGAATACCACAGGGCGCAATGTGCG
>OTU_95
TTAAGTTCAGCGGGTAGTCCCGCCTGATTTGAGGTCAAGTTTGAAGTGATT
GTCTCTTGCGAGACGGTTGGAAGCGATTCCCGTATGTTCGCTAAACCGAG
GTGTAGATGACTATCACACCAAGGCCGCAAGGGCTTCGCTAATGTATTCA
AGGAGAGCGGATCGGCCAGGGACCCGCAAGCTCCCAAATCCCAGCCCAA
CGCCTTCCGAAAAAGGTGGAGGGTGGAGGAGTTCACGACACTCAAACAG
GCGTGCCCCTCGGAATGCCAAGGGGCGCAAGGTGCGTT
>OTU_102
TTAAGTTCAGCGGGTATCCCTACCTGATCCGAGGTCAACCTGAAAAAAAT
ATTTGGTGGTTTGACGGCGGGAGACCCCAAGGGCTCCGAGGACGTTATTG
AAGTAATTCTACTACGCCCAGAGACCGAGTGGCTCCGCCGAAGGGTTTGG
GGCGCGGCCGCCGCTCTCGCGGGGCCGGACGCCCAATACCAAGCAGTGCT
TGATTGGTGAAATGACGCTCGAACAGGCATGCCCCTCGGAATACCAAGGG
GCGCAATGTGCGTT
>OTU_106
TTAAGTTCAGCGGGTATCCCTACCTGATCCGAGGTCAAAGTTAAAAAAAG
GCTTATGGACGCAAGTATTCTTGGGTAGAATCGCAAAATGTGCTGCGCTT
CAATACCAAAACACTGGCTGCCAAATCGCTTTAAGGCGAGTCCAAACGCA
GAGGAGAGGACAAACACCCAACACCAAGCAGAGCTTGAGGGTACAAATG
ACGCTCGAACAGGCATGCCCCATGGAATACCAAGGGGCGCAATGTGCGTT
>OTU_111
TTAAATTCAGCGGGTAGTCCCGCCTGACCTGGGGTCGCGTTGGAAGCGTC
GCTAGCGCGACACAGCAGGGTCCAAGGAGCACACGATGAGCGACGCGGC
ACGCACGACGGGACACGAGGGTTTGACAACCACCGATTGTCGTGGCGCGC
GTCGCCGAGGACTCGCTTTTAGGCCAACCGCATGCATGAGCTCACGGGAG
GCCAATTTCTGCCCCACAGGCCCCCCTCGTCCCTTTGCAAGGAGATGGGGT
TGGGGGCAACGATGCGTGACACCCAGGCAGACGTGCCCTCGGCCAGGTG
GCTTCGGGCGCAACTTGCGTT
>OTU_115
TTAAGTTCAGCGGGTAGTCCTACCTGATTTGAGGCCAGATGTCAAGAATG
TGGTAGCCTCTCTAGGTGTTGTGCACTTTGAGGCGAGATCAAGCAATTCA
GTCGGCCACCCTTGCGAGTGTCCTCAGCGAAATAATTTATAACGCCAAGT
CAAACCGGCCGGCAGGCAGGATCACTACAATACATTTAAGGTGAGCGGG
GATCATCTCCACGCAAGCACCCAAGTCCCAGCCAGACCCTTGGACAAAAG
CCCAAAGGTAAGGTGGATGATTTCATGACACTCAAACAGGCATGCTCCTC
GGAATACCAAGGAGCGCAATTTGCGTT
>OTU_117
TTAAGTTCAGCGGGTTTTCTTGCTCGATTTCAGACCAAGTAATCAGAACAC
GCATCAAAGTAGACGCGCATCCTGTATCATTGATGGGGTGGATGACCCGA
TATCACCAGTCCAAGTCACAAGAGAAATAGAAATCTGTTGCTCTTGTGTTC
CCGGCATGGATTTACCGTGACACACACAATCAAGTTAATGCGGCTGCCCG
AAGGACAGTTTCCGCACACATCCAAGCCCCACACGGTCCGAAAACCGTAT
GGTTTTTGAGAATTTAATGATTCTGAAACGAGCATGCTCCCCGGAATACC
AGGAAGCGCAATGTGCGTT
>OTU_120
TTAAGTTCAGCGGGTATTCCTACCTGATCCGAGGTCAACCTGAAAAAAGT
TTTAGAGGCGACCGGGTCACTGGGCCCTTGAAACTCTACGCGTGCTCCGG
AAAGCTCAGTGCGAAGAGGGTCCCTGGGACGCCGTCATTGTCTTTGAGAC

CAGTTGGCCATGGAGGTGGTCCAACGCGGTCCAACACCAAACCCGGGGGC
TTGAGAGAGGATAATGACGCTCGAACAGGCATGCCCTTCGGAATGCCAAA
GGGCGCAATGTGCGTT
>OTU_125
TTAAGTTCAGCGGGTATCCCTACCTGATCCGAGGTCAACCTTTGAAATGTG
TTTAACGGCGCGAATCGTCCGTATCCCCAAAGCGGAAACATAAAGTGCCA
CAACGCTTAGAGACGAACGACTCAGCCGGAGACATTGAGGCGCGCGAAC
GGGCGCGGCGCCCAATACCAAGCGAGGCTTGAGTGGTGTAATGACGCTCG
AACAGGCATGCCCCTCGGAATACCAAGGGGGCGCAATGTGCGTT
>OTU_128
TTAAGTTCAGCGGGTATCCCTACCTGATCCGAGGTCAACCTTAAGGAGTT
GGGGTTAAGGCAAGTGGGTGTTAGGGGTCTGTCGCGAGGAGTATTACTAC
GCGTAGAGCCTAACAGCACCGCCACTGATTTTGAGGCCCGCCCAGAGGCG
AAGCCCAAGACCCTCTATGGAGAGGAGTAGGTCAAAATGACGCTCGAAC
AGGCATGCCCCTCGGAATACCAAGGGGCGCAATGTGCGTT
>OTU_133
TTAAGTTCAGCGGGTATCCCTACCTGATCCGAGGTCAAGAGTGTAAAAAT
GTACTTTTTGGACGTCATCGTTATGAGTGCAAAGCGCGAGATGTACTGCG
CTCCGAAATCAATACGCCGGCTGCCAATTGTTTTGAGGCGAGTCTGCGCG
CAGAGGCGAGACAAACACCCAACACCAAGCAAGGCTTGAAGGTACAAAT
GACGCTCGAACAGGCATGCCCCATGGAATACCAAGGGGCGCAATGTGCGT
T
>OTU_137
TTAAGTTCAGCGGGTAGTCCTACCAATCTGAGGCCGATGAATTGAAATGA
AATCCCTTCCTCCTTGCAGAGGCATGGGCGGGGTTCAGAAGCACGCCAAG
CAGCAAAGTCGTCGCGTCCAGCTCTCTCCCTTCCTTCGCCTCCGAAGTCCT
GATATTATCAAAACCCCGGCAGGGAAGAGAAGGGCGAAAAAGCGAGCTT
TCGTCCGTCTTTGCCTATCAAATGGATGCGCTAATGCATTTCGAGGGAGCC
ATGGTAACTGGCAAAAAAAAAGAACCCTCAATACCGATCCGCCAACTCTT
ACTGAAAAAAGCTGTCGTTCGAAACAATTCGCGGCCCTCAAACAGGCATG
CTCCCCAGATTAGATCTGCCGGGAGCGCAAGGTGCGTT
>OTU_143
TTAAGTTCAGCGGGTATCCCTGCCTGATCCGAGGTCAAACTGTAGTAATA
AAACTATGCGGTAGACAACGCCCCAAAAAGACTTGAGAGTGAAAATGTTT
TACTGCTACACTCTAAGCTGGGGGTTAAATCGCCGTCTCTATTTAAGGCAC
GTTAGGACCCTTCTTTATTATAAACAAGGTAAACGATGCCCAATACCAAG
CTAAGCTTGAGTGGTAGAAATGACGCTCGAACAGGCATGCCCCCCGGGAT
ACCGAGGGGCGCAATGTGCGTT
>OTU_145
TTAAGTTCAGCGGGTAGTCCTGCCTGATTTGAGGTCTAGAGTGTAAATGA
CTGATTAAAGTCGCATTAGAAGCGTCAAAGACACAATTGAATTTCGAACG
TCCTCAGCGAATAGTATTATTACGCCGAGTCAAGCCGTTTTCATATTCATA
TGAACTTTGGGTCGCTCGTATATTTCAGCTGAGCCGGCTCAAGTAAGAGG
TCCGACAGACAACCATAATCCAAGCCCGCAACTCATCTCATTACAAAATA
AGGGGGTTGAGAGTTTCACGACACTCAAACAGGCTTACTCTTCGGAATAC
CAAAGAGTGCAAGGTGCGTT
>OTU_148
TTAAGTTCAGCGGGTATCCCTACCTGATCCGAGGTCAAAAGGTTGAAAAA
AAAAAGGCTTCATGGACGCGCGACCGCGGCTGGACAAGAGCGCAAATAA
TGTGCTGCGCTCCGAAGCCAGTAGGCCGGCTGCCAATGTTTTAAGGCGA

GTCTCGGGGGAGAGACAAAAGACGCCCAACACCAGGCAAAGCTTGAGGG
TACAAATGACGCTCGAACAGGCATGCCCTTTGGAATACCAAAGGGCGCAA
TGTGCGTT
>OTU_172
TTAAGTTCAGCGGGTAGTCCCGCCTGATTTGAGGTCAAGTTGGTAGTAGTT
GTCTCTTGCGAGACGGTTGGAAGCAAGTCCCCGTATGTTCGCTAAGCCGA
GGCGTAGATGACTATCACACCAAGGCCGCAAGGGCTTCGCTAATGTATTC
AAGGAGAGCGGATCGGCCAGGGACCCGCAAGCTCCCAAATCCCAGCCCA
ATGCCTTCCGAAAAAGGTAGAGGGTGGAGGAGTTCACGACACTCAAACA
GGCGTGCCCCTCGGAATGCCAAGGGGCGCAAGGTGCGTT
>OTU_195
TTAAGTTCAGCGGGTATCCCTACCTGATCCGAGGTCAACCATAGAAAATT
GGGGGTTACTGGCCAGCATCCGCCGGGACCCTATAGCGAGAGAGATTACT
GCGCTCAGGGCCCGACGGCACCGCCACTGATTTTAAGACCCGCCGGGTTA
GCGGCGAAGCCCAAGACCAAGCTGTGCTTGAGGGTTGAAATGACGCTCGA
ACAGGCATGCCCCTCGGAATACCAAGGGGCGCAATGTGCGTT
>OTU_260
TTAAGTTCAGCGGGTATCCCTACCTGATCCGAGGTCAACCGTGAGATTGG
GGGTTGATGGCAAGCATCCACCGGGAACCCTATAGTGAGGAGTATTACTA
CGCTTAGAGCCAGATGGCGCCGCCACTGATTTTAAGGGCCGCGGGACCGC
GGACCCCAACACCAAGCCGGGCTTGGGTGGTCATAATGACGCTCGAACAG
GCATGCCCCTCGGAATACCAAGGGGCGCAATGTGCGTT
>OTU_94
TTAAGTTCAGCGGGTAGTCCTACCTGATTTGAGGCCAGATGTTATGAATAT
TAATCCGAAGATCAATGGATTAGAAAGCGGTCTTTAGTCCTGCAACAGGG
CCATCCGAAGATGTCCTTAGCGAAATACTTATTACGCCAAGTCAAACCAT
GTCGCGAGACAGATCCAGCTATTACTTTTAAGACGAGCCGACTTGACATC
GGCAAACGTCCAAATCCAAGCCAAAGAAAGGTCAAATAACCAATCTAAG
GTTGAGGGTTTTCATGACACTCAAACAGGCATGCTCCTCGGAATACCAAG
GAGCGCAAGGTGCGTT
>OTU_103
TTAAATTCAGCGGGTAGTCCCGCCTGACCTGGGGTCGCGTTGGAAGCGTC
GCTAGCGCGACACAACAGGGTCAAGGAGCACACGATGAGCGACGCGGCA
CGTACGACGGGACACGAGGGTTTGACAACCACCGATTGTCGTGGCGCGCG
TCGCCGAGGACTCGCTTTTAGACCAACCGCATGCATGAGCTCACGGGAGG
CCAATTTCTGCCCCACAGGCCCCCCTCGTCCCTTTGCAAGGAGATGGGGTT
GGGGGCAACGATGCGTGACACCCAGGCAGACGTGCCCTCGGCCAGGTGG
CTTCGGGCGCAACTTGCGTT
>OTU_104
TTAAGTTCAGCGGGTAGTCCTGCCTGATTTGAGGTCAGATTCAAGATGGA
GTCAGTCCCCCAAGAGGGACGATTCGAAGCCGAAGCCACACGGCTCCGA
GACGGAATTATCGAGACGTAACAAAAACCTATCACGCCACGGACCGCCCG
AAGCCAACCTCGAGCTAATGCATTCCAGAGGAGTCGACCCCCCTCAAGCC
CCCGGCAAGGGAGGCAAGAGGCCAACAACCCCCAAATCCAAGCCTCGCC
TCGCAATTTTCAAAGCGAGACAAAGTTGAAGACTTCACGACACCCAAACA
GGCATGCTCCTCGGAATACCAAGGAGCGCAAGGTGCGTT
>OTU_105
TTAAGTTCAGCGGGTATCCCTACCTGATCCGAGGTCAACCTAGAAAAATG
GGGGGTTTTGGCAAGTAACCACCGGAACCCTATAGCGAGAGAAATTACTA
CGCTTAGAGCCAGACGGCACCGCCACTGATTTTAAGGGCCGCGAACACGC

GAACCCCAATACCAAGCTAGGCTTGAGTGGTTATAATGACGCTCGAACTG
GCATGCCCCTCGGAACACCAAGGGGCGCAATGTGCGTT
>OTU_109
TTAAGTTCAGCGGGTGCCCTTGCCTGAACTCAGGTCGAATGGTTTGGCAG
CGGTGGCGATTGAGCCACACACGCCGTCTCCTGCTGGGCGTCCAGCGGGT
CCCGGGGTCCAGCAGCGGCATGCGATCCTTCCGATGGAAGGGCTCACCTA
CCGGTCTGTTGTTCCCACGTCAAAGGGGATCCCGCTCAAGCCTCTGCGCTT
CAGCTGACCCGGTCTGTTAGGACCGAGACGGCCAGATCCACTCTACCCAC
TGAAAGGGGAAAGCGAAGGTAAAACCGACACTGAGGCAGGCATGCTCTC
GGCCGAAGCCTCGAGCGCAATTTGCGTT
>OTU_110
TTAAATTCAGCGGGTAGTCCCGCCTGACCTGGGGTCGCGTTGGAAGCGTC
GCTAGCGCGACACAGCAGGGTCAAGGAGCACACGATGAGCGACGCGGCA
CGCACGACGGGACACGAGGGTTTGACAACCACCGATTGCCGTGGCGCGCG
TCGCCGAGGACTCGCTTTTAGGCTAACCGCATGCATGAGCTCACGGGAGG
CCAATTTCTGCCCCACAGGCCCCCTCGTCCCTTTGCAAGGAGATGGGGTTG
GGGGCAACGATGCGTGACACCCAGGCAGACGTGCCCTCGGCCAGGTGGCT
TCGGGCGCAACTTGCGTT
>OTU_113
TTAAGTTCAGCGGGTATCCCTACCTGATCCGAGGTCAACCTTGAGGTTATT
TGGTTTTTGGAAGCGGGCAGACGACGGAATCCTCAGAAAGCGGGATATGT
TACTACTACGCTTGAGGCTCCGCGCCGCCGCCGAGGTCTTTGGGGCCCGTC
CGCAGCAGGGACGAAGACCCAATGCCAAGCAGTGCTTGATGGTCTATAAT
GACGCTCGAACAGGCGTGCCCTCCGGAATGGCCGGAAGGCGCAATGTGC
GTT
>OTU_114
TTAAGTTCAGCGGGTATCCCTACCTGATCCGAGGTCAACCTTTAAAAGTTT
GGTGTTTAGTGGCAAAAGAGTCAGACCGGTACTTATAAGACGTTGAGAAA
TTCTACTACGCCCAAAGCCGGTGAGGCTCCGCCGAGGTGTTTGGGGCACG
CCCACCTTGCGGGGGACGATGGCCTAATACCAAGCAGTGCTTGAGTGGTG
TAATGACGCTCGAACAGGCATGCTCCCCGGAATACCAGGGAGCGCAATGT
GCGTT
>OTU_116
TTAAGTTCAGCGGGTAGTCCTACCTGATTTGAGGCCAAAATAGAAAAATT
ATCTCGAAAGAGATAGGTTAGAAAGCAGACTTCATATTACGAGAGTTCAA
AATGTTACTTAACAACTCTGCTGAAGTCCAGCTAATTCATTTAAGGTAAGC
TTGTGTTTGTAACAAACCGCAAGCAGACCCAAGGACCCGACTTGTCATAT
TTTAATTAAAAAATATAAAAGCGGAGATATTCAAGACTCTCAAACAGGCA
TGCTCCAAGGAATACCAAGGAGCGCAAGGTGCGTT
>OTU_119
TTAAGTTCAGCGGGTAGTCCTGCCTGATTTGAGGTCAAAATGAATAGAGT
GGGGCTCGGAGCGCACACCACGGGCAAGCCTAGGCTGGCCGACGAGGGC
TGTACAACGTCGGGGCCGTGAGACGGCTGCCGTTGGGCGCTGGCGACAGA
GCGCTGCATTTCGGCTCGCGACGTCGTCGCACCTTCTTGCGAAGACACGA
GTCGGTCGGCATGGGAGCCATGATCCAAGCCGCCGCCTCTCGTGAGAGAG
GCGGGGTTGAGAGGGGAGCGCGACACTCAAACAGGCATGCTCCGCGGAG
TACCACGGAGCGCAAGGTGCGTT
>OTU_121
TTAAGTTCAGCGGGTAGCCCTACCTGATCCGAGGTCAACCTGTAAAAATT
AGGGGGTTCTGGCAAGCAACCAGGGGAACTCCATCGCGAGAAGAATTAC

TACGCGTAGAGCCACACCGGCACCGCCACTGATTTTAGGAGCTGCGGAAT
CGCAAACTCCAATACCAAGCCAGGCTTGAGTGGTTATAATGACGCTCGAA
CAGGCATGCCCTGCGGAATACCACAGGGCGCAATGTGCGTT
>OTU_122
TTAAGTTCAGCGGGTATTCCTACCTGATCCGAGGTCAACATTTAGAAATTC
ATCAATTGACTGTCAACATCCCGCCAGCTAGACGACGTGTGTGCGCCTGT
GGTTGAATATCACCATCGCTGATTTTGAGGCATGCCTTTAGGCGTTGCCCA
ACGCCAAAGAGTCATCTTTGAGGGGTGTAATGACGCTCGAACAGGTATGC
CCCTCGGAATGCCAAAGGGCGCAATATGCGTT
>OTU_123
TTAAGTTCAGCGGGTATCCCTACCTGATCCGAGGTCAAAAGTTAAAAAAG
GCTTATGGACGCAAGTATTATCGGCTAGAATCGCGAAATGTGCTGCGCTT
CAATACCAAAACACTGGCTGCCAATTGCTTTAAGGCGAGTCCAAACGCAA
AGGAGAGGACAAACACCCAACACCAAGCAGAGCTTGAGGGTACAAATGA
CGCTCGAACAGGCATGCCCCATGGAATACCAAGGGGCGCAATGTGCGTT
>OTU_124
TTAAATTCAGCGGGTTGTCACGCCTGACCTGGGGTCACGTTGGAAGCGTC
GCTAGCGCGACACAGCAGGGTCAAGGAGCACATGATGAGCGACGCGGCA
CACACAACGGGACACGAGAGTTTGACAACCACCGATTTTCGTGGCGCGCG
TCGCCGAGGACTCACTTTTAGGCCAACCGCATGCATGAGCTCACAGGAGG
CCCCCTCGTCCCTTTGCAAGGAGATGGGGTTAGGGGCAACGATGCGCGAC
ACCCAGGCAGACGTGCCCTCGGCTAGGTGGCTTCGGGCGCAACTTGCGTT
>OTU_126
TTAAGTTCAGCGGGTATCCCTACCTGATCCGAGGTCAACCTTTGAAAGATT
TAACGGCCGATCCAACTCGAACTCCACTGCGAAATAGTTGCCACAACGCT
GGGAGACGGGTCAAACAGCCGGAGACTTTGAGGCGCGCGAGAGACTCGC
GACGCCCAATACCAAGCGAGGCTTGAGTGGTGAAATGACGCTCGAACAG
GCATGCCCCCCGGAATACCAGGGGGCGCAATGTGCGTT
>OTU_127
TTAAGTTCAGCGGGTATCCCTACCTGATCCGAGGTCAACCTTGTAAAAATT
GGGGGTTGTTTTAGCAGGCCGCCGACCAAGCCTAACAAGCGACGAGTATT
ACTGCGCTTGAGACTGGACGGAGCCGCCACTGATTTTGAGGCAGGCCCAT
GAAGGGCGAGCCCAATACCAAGCTGAGCTTGAGGGTTGTAATGACGCTCG
AACAGGCATGCCCCCCGGAATACCAGAGGGCGCAATGTGCGTT
>OTU_130
TTAAGTTCAGCGGGTAGTCCTACCTGATTTGAGGTCAGATTTCAATGTTTA
GTTGACCGCCACTCAGAAGGCAGGTCGTGGAGGGGCACAGAAGGGGAAC
ATCTCGTTAGAGCGAGGATGGTACAACGCCCAATCGAGTCCGACCCGCAG
GCCCAACCGATACATTTGAGGGGAGCCGCCTCACGGCAGCAAGCCCCCAG
CTCCAACCCACAGCTGTACAAAACAGGAGGGTTGATGACTTCAAGACACT
CAAACAGGCATGCTCCTCGGAATACCAAGGAGTGCAAGGTGCGTT
>OTU_131
TTAAGTTCAGCGGGTAGTCCTACCTGATTTGAGGTCAAAATGTCAAGTGG
GGTTTTTCCAAGTCAATGGAAAAGGTTGGGCATAGACGTTAAAGCAATCC
AGCCACGGTGATCATTATCACACCAAAAGGCCGATTCATGCACACGCATT
TGCCAATGCATTTCAGAGGAGCCGACTTCACAGCCGGCAGAGCCTCCACG
ATCCAAGCTCACCCTTGAAAAAAATCAAAGGGAGTTGAGAATTTAATGAC
ACTCAAACAGGCATGCTCCTCGGAATACCAAGGAGCGCAAGGTGCGTT
>OTU_134

ATAAGTTCAGCGGGTATCCCTACCTGATCCGAGGTCAAAAATAGAAAATA
GCTTATGGGAGCTGATCCATTGTCCTCGAGTTCGCGTTTCTGCTGCGCTCG
AAGCCAATGTACCGGCCGCCAATGAATTTAAGGCGAGTCCGCGCCGAGGC
GGGACAGACACCCAACACCAAGCTGAGCTTGAAGGTTTAAATGACGCTCG
AACAGGCATGCCCTAAGGAATACCAAAGGGCGCAATGTGCGTT
>OTU_135
TTAAGTTCAGCGGGTATCCCTACCTGATCCGAGGTCAACACTTAGAAAAT
ACTCCGAAGAGCAGTGGTTAAAGACCGCCGCCCGCAGATACACGCCGCGT
ACATGCTTGGTTAAAAGCTCCTCGTACCGGTTCCATCTGGGACGCCGTCAC
TATCTTTGAGGCCGATTTGGTGACAAATCCAGCCCAACACCAAGCCAGAG
CTTGAGGGGTGATAATGACGCTCGAACAGGCATGCCCTTCGGAATACTAA
AGGGCGCAATGTGCGTT
>OTU_136
TTAAGTTCAGCGGGTAGTCCTACCTGATTTGAGGCCATATGTCAAAGTAAT
ACTATAAGTAACACCAGAGATGCACTTAAAGTAAGGTTTAGTAAGCAGAC
AAAAGTCAAGTCCTGGCCATCCGAAGATGTCCTTAGCAAAATACTTATTA
TGCCAAGTCAAACCAGTCAAAGACAGATCTAGCTAATACTTTTAAGGTGA
GTCAGTTCATCACTGGCAAACACCCAAATCCAAACTCAAGCAAGGATAAA
TCCAAAACTTGGGTTTGAGAGGTTTATGACACTCAAACAGGCATGCTCCT
CGGAATACCAAGGAGCGCAAGGTGCGTT
>OTU_138
TTAAGTTCAGCGGGTATCCCTACCTGATCCGAGGTCAAAAGCAAAAGTAT
AGACTTCATGGACGCAAGCGTTCAGGTTCAGAGGCGCAAATTGTGCTGCG
CTCCCAGACTAGTACGCCGGCTGCCAATTGCCTTAAGGCGAGTCCACGCA
CAAAGCGGGACAAACACCCAACACCAAGCAGAGCTTGAAGATACAAATG
ACGCTCGAACAGGCATGCCCCATGGAATACCAAGGGGCGCAATGTGCGTT
>OTU_142
TTAAGTTCAGCGGGTATCCCTACCTGATCCGAGGTCAACCTGTAAAAAAT
TGGGGGTTCTGGCAGGCCACCGGGGGAACTCAATCGCGAGGAGATTTACT
ACGCGTAGAGCCCACCGGCACCGCCACTGATTTTAGGGGCTGCGGAACCG
CAAACCCCAACACCAAGCCAGGCTTGAGTGGTTATAATGACGCTCGAACA
GGCATGCCCTGCGGAATACCACAGGGCGCAATGTGCGTT
>OTU_146
TTAAGTTCAGCGGGTAGTCCTACCTGATTTGAGGTCAGATGACATGAGTTT
GTCCGAAGACGATTGGAAGCGACCCTCGCTCGAGCCAACGTAACACGGCG
CCACAGTCACGAAGCGTTTCTTATCACACCGTTGTTGAACTCGGGCGGCG
GACCAGCTGATGCATTTTCAAGACGAGCCGAAGCGTGAGCCACGGCAGCG
TCCAAAATCCAAACCCGCTTCACAAAACTGAAACGGGATTGAGAGGTTCA
CGACACTCAAACAGGCATGCTCCTCGGAATACCAAGGAGCGCAAGATGC
GTT
>OTU_149
TTAAGTTCAGCGGGTAGTCCTACCTGATTTGAGGTCAGATTTCAAAGTTTA
GTTGACCGCCGCTCAGAAGGCAGGTCGTGGAGGGGCACAGAAGGGGAAC
ATCTCATTAGAGCGAGGATGGTACAACGCCCAATCGAGTCCGACCCACAG
GCCCAACCGATGCATTTGAGGGGAGCCGCCTCACGGCAGCAAGCCCCCCA
GCTCCAACCCACAGCTGCACAAAACAGGTGGGGGTTGATGACTTCAAGAC
ACTCAAACAGGCATGCTCCTCGGAATACCAAGGAGCGCAAGGTGCGTT
>OTU_150
TTAAGTTCAGCGGGTATTCCTACCTGATCCGAGGTCAATTTTTCAAAAAAT
GGGGGGAGTTTTCTGGCTAGAAGTCCCACTAGTCTTTACAAACGAGGTAT

AAATTACTACGCTCAAAATTCTAGCGAGCCCGCCACTGTATTTCAGGGCA
TACCCTTTGACGGGTAAAGCCCCAACACCAAAGACGAAGCTTTGAGGGTT
GAAATGACGCTCGAACAGGCATGCCCGCTGGAATACCAGCGGGCGCAAT
GTGCGTT
>OTU_151
TTAAGTTCAGCGGGTATTCCTACCTGATCCGAGGTCAAATTTCAGAAAGTT
TGGGGGGTTTTACGGCAAGAAGTCCCACTAGTCTTTAAAAACGAGGTATA
ATTACTACGCTCAAAATTCTAGCGAGCCCGCCACTAAATTTCAGAGGGTA
CCCTGTTACAGGTATTCCTCCAACACCAAAAATACAATATTTTTGAGGGTT
GAAATGACGCTCGAACAGGCATGCCCGGTGGAATACCACCGGGCGCAAT
GTGCGTT
>OTU_152
TTAAGTTCAGCGGGTATCCCTACCTGATCCGAGGTCAAAAGTTAAAAAAG
GGCTTGTGGACGCAAGTATTTGATAAGAATCGCGACTTGTGCTGCGCTTC
AATACCAAAACACTGGCTGCCAATTATTTTAAGGCGAGTCCAAACACTAG
GGAGAGGACAAACACCCAACACCAAGCAGAGCTTGAGGGTACAAATGAC
GCTCGAACAGGCATGCCCCATGGAATACCAAGGGGCGCAATGTGCGTT
>OTU_154
TTAAGTTCAGCGGGTAGTCCTGCCTGATTTGAGGTCAAAATGAATAGAGT
GGGCTCGGAGCGCACACCACGGGCAAGCCTAGGCGGGCCGACGAGGGCT
GTACAACGTCGGGGCCGTGAGACGGCTGCCGTTGGGCGCTGGCGACAGA
GCGCTGCATTTCGGCTCGCGACGTCGTCGGTTCCTCGCGAGAGGGGCCCG
AGTCGGTCGGGATTGGAGCCATGATCCAAGCCGCCGCTTCTCGTTAGAGA
GGCGGGGTTGAGAGGGGAGCGCGACACTCAAACAGGCATGCTCCGCGGA
GTACCACGGAGCGCAAGGTGCGTT
>OTU_155
TTAAGTTCAGCGGGTATCCCCGCCTGATCCGAGGCCAAACCGTGTAAATG
AGTTTCAGAGGCAGCCGTCCCGATGGGCACGCGGAGCGAGGAACGAATT
CTTGCTACGCTCGCGGCCAGCGGGGGGCGCCTAGGGGTTTGGGGTCTGGT
AGCGTACCACGACCCAACATCAAGCAAGGCTTGGGGGGTGTAATGGCGCT
CGGACAGGCGTGCCCCCCGGAATACCAGGGGGCGCAATGTGCGTT
>OTU_156
TTAAGTTCAGCGGGTATCCCCGCCTGATCCGAGGCCAAACCGTGTAAATG
AGTTTCAGTGGCAGCCGTCCCGATGGGCACGCGGAGCGAGGAACGAATTC
TTGCTACGCTCGCGGCCAGCGGGGGGCGCCTAGAGGTTTGGGGTCTGATA
GCGTACCATGACCCAACATCAAGCGAGGCTTGGGGGGTGTAATGGCGCTC
GGACAGGCGTGCCCCCCGGAATACCAGGGGGCGCAATGTGCGTT
>OTU_158
TTAAGTTCAGCGGGTATCCCTACCTGATCCGAGGTCAACCTTTGTGTGGGG
GTTTAACGGCGTCAAACGGTCCAATCCCTCAAGCGAATTGAGTTGCCACA
ACGCTTTGAGACGGACGGCTCAGCCGGAGACATTGGGGCGCGCCGGCAC
GCGGCGACGCCCAAGGCCCAGCGAGGCTGGAGTGGTGAAATGACGCTCG
AACAGGCATGCCCCCCGGAATACCAAGGGGCGCAATGTGCGTT
>OTU_160
TTAAGTCCAGCGGGTATCCCTACCTGATCCGAGGTCAACCTTTAGAGTGG
GCACGAGTGCCCGGTTTCACGGCCGAGGCCCGCGGTTGCTGGACGTCTGG
GGTCTCCCCCTTTCTGATACGCCCGGCGAACGCGAGGTCCCGCCGACTCTT
TTGGGGCACGCCCTGGCCGAGGCCGGGGGCGAAGCCCATCGCCAAGCTG
GTGCTTGATGGTTGTAATGACGCTCGAACAGGCATGCCCCCCGGAATGCC
GAGGGGCGCAATGTGCGTT

>OTU_161
TTAAGTTCAGCGGGTAGTCCCGCCTGATTTGAGGTCAAGTTTCGATGAAGT
TGTCTTTCGACGGTTGGAAGCAGGTCCCCCACAATCGCGGTGTACCCACG
GCGTAGACAATTATCACACCGGGGGGTGGCACCACAAGGGTCTCGCTAAT
GCATTTGAGAGGAGCCGATCCGCGAGGACCAGCAAAGCCTCCAAGTCCA
AGCCCACGCGCTTTCACAAGAAAAGCGCAGGGTTGAGAATTTCACGACAC
TCAAACAGGCGTGCCCTTCGGAATACCAAAGGGCGCAAGGTGCGTT
>OTU_165
TTAAGTTCAGCGGGTAGCCCTGCCTGATTTGAGGCCACATCCTAAAGGTG
TGAGTACGAATTAGAAGCACCACTTATCGATTGAAAGGAAGCGTCCTCAG
CGAAATTGTTATTACGCTGAGTCAAGCCGCTTATTTCAAACAGGGTTGCTC
TTGTATTTCAGCCGAGCCGGTGTAAAGCCAGCAGCAGCCATTATCCGAGC
CCACAGCACAGTCCCATTACAAGACAGGCGGGGTCGAGGGTTTCGTGGCA
CTCAAACAGACATACTCTTCGGAATACCAAAGAGTGCAAGGTGCGTT
>OTU_173
TTAAGTTCAGCGGGTAGTCCCACCTGATTTGAGGTCTAAATGATATATATA
TTAGGGTTCGGAAGCTATCTATCAAAAGTTCAAATGGCTGTAGTGTTTTTA
AGGCACCACACTCCACAATGAATAATAAATACTACACCAAGTATATCCAT
TTATTTTCTCAATAAAAGTACTTATATATTTAAGGTGAGCCAATAACGGCA
ACACCCAACATCCATTCCAACTTCTTAACTTAATAAAAAATTGGAATGAG
AGGGCTTCATGACACTCAAACAGGTGTACCTTTCGGAATAACCAAAAGGA
GCAAGGTGCGTT
>OTU_180
TTAAGTTCAGCGGGTAGTCCTGCCTGATTTGAGGTCAAAAATGTAAGAGT
GGGTTCGGAGCGCACACCACGGGCAAGCCTAGGCTGGCCGACGAGGGCT
GTACAACGTCGGGGCCGTGAGACGGCTGCCGCTGGGCGCTGGCGACGGG
CGCTGCATTTCGGCTCGCGACGTCGTCGGTTCCTCGCGAGAGGGGCCCGA
TTCGGTCGGAGTGGGAGCCATGATCCAAGCCGCCGCTTCTCGCAAGAGAG
GCGGGGTTGAGAGGGGAGCGCGACACTCAAACAGGCATGCTCCGCGGAG
TACCACGGAGCGCAAGGTGCGTT
>OTU_182
TTAAGTTCAGCGGGTATCCCTACCTGATCCGAGGTCAACCTTAGGTGATG
GGTTCTAGGGGCAAGCAACCGCCGAGGCCTCTGAAGCTAAAGATTTTACT
ACGCTTGAGGCTCGGGGCCACCGCCGAGTGCTTTAGAGCGCGCCCCCCCG
CTAAGGGGGGACGGACGCCCAATACCAAGCTGGGCTTGATGGTTGATAAT
GACGCTCGAACAGGCATGCCCCTCGGAATACCAAGGGGCGCAATGTGCGT
T
>OTU_184
TTAAGTTCAGCGGGTAATCCCACCTGATTTGAGGTCTAAAAGTTTGATAA
ACTGGGGTTAGGAAGCTATCTATTGAAAACTCAAATGGCTGTAGTAACAA
AACTACATTCCTCAATGAATAATAAATATTACACCAAGTATTTCTCCATTT
GTTTATTCAATAAAGGTACTTATAAATTTAAAGCGAGCCAATGACGGCAA
CGCTCAACATCCATTACACATTTCCTTAATCAAATAAGGAATTGGAATGA
GAGGGTTTCATGACACTCAAACAGGTGTACCTTTTGGAATACCAAAAGGT
GCAAGGTGCGTT
>OTU_203
TTAAGTTCAGCGGGTAGTCCCACCTGATTCGAGGTCAATGTTGAAAGGTC
GTCGCCGCAAAGGACAGACAGATTGGAAGCGATCCAAGGCACTCTTGGCC
ATCCGCCCAAAAGCAGATGTCCTCAGCGGTCATAACTTATCACGCCGAGT
AGAACCAAAGATACATCATGGGTCAGCTAATGCATTTAAGACCAGCCGCC

ACAACCATAAAGTCATGCGCGGCAAAGTCCAAGTCCAAGCCAAGATAGC
AACAAAGCATCCGACTTGAGGTATTTCACGACACTCGAACAGGCGTGCTC
CCCGGAATACCAGGGAGCGCAAGGTGCGTT
>OTU_218
TTAAGTTCAGCGGGTATCCCTACCTGATCCGAGGTCAGAACGTTGGTGCG
TGGGGGGGGTCTCGAAGCGGGCGGGCGCCCGAGCCCGGACAGACGACAT
CTTGCGATCCGCTACGTCTGGGGCTCGTCGCCGCCGCCGACACATTCAGG
GCGCCCCCGGGGGGACCCGGGGGAGACGCCCAAGGCCCGCCCCCCGGCG
CTTTCACGCCGGGGGCCGGAGGGGTCGTGACCGACGCTCGAACAGGCATG
CCCCGCGGAATACCACGGGGCGCAATGTGCGTT
>OTU_227
TTAAGTTCAGCGGGTATCCCTACCTGATCCGAGGTCAACCTGTAAAAATG
GAGGGGTTGCTGGCGACCGTCCGCGCGATAACAATCAGCTTTTAGTAATT
ACTACGCTGAGTACCTTGCGGCGATGCCACTGATTTTGAGGGCCGCGAAA
CCGCGTGCCCCAACACCATGCTAGGCATGATTGGTTTAAATGACGCTCGA
ACAGGCATGCCCCGCGGAATACCACGGGGCGCAATGTGCGTT
>OTU_231
TTAAGTTCAGCGGGTGTTCTTGCCTGACCTCAGGTCGAAAGCCAAAAGGC
GTCCTGCACATCGCATTTGGGAGCACAGCGGGCAACGAAGGCATGCAACC
GGCAAAGGTCACCTACCAAATCGGAACTCGCCTTTCCTCGAAAGGAAAGC
GGCCCCCAAAGCGTGAATATATTTCAGCTGACCGGCCACCCAAAGGGCAA
ACCGGACGGCCAGATCCAACTGCCTTTCATCGGAATTCACCAATGAAATT
TAGTTGAGGGTAGTAGCCGACGCTGAGGCAGACATGCTCTTGGCCGAAGC
CTCGAGCGCAATTTGCGTT
>OTU_253
TTAAGTTCAGCGGGTATCCCTACCTGATCCGAGGTCAACCTTGAGGTGAG
ATGTTCTCGAGGCAGAACGGCTACAACCTAGAAAGCTAAGAATTTCTACT
ACGCTTGAGGTGTATAACCGCCGCCAAAGGGTTTAAGGCGCGTCCGCTAG
GGACGACACCCAATGCCAAGCAGAGCTTGATGGTTGATAATGACGCTCGA
ACAGGCATGCTCCCCGGAATACCAGGGAGCGCAATGTGCGTT
>OTU_264
TTAAGTTCAGCGGGTAGTCCTACCTGATTTGAGGTCAAGAAGTTCACTAA
ACATTGTCCATAACGGACGATTAGCAGCTGAACTAGTCAGAGAGCAATCC
ATCACAGTGTAGATAATTATCACACTAGTGACGGGACTGCAAACGGTTCC
GCTAATGTATTTCAGGAGAGCTGACCTTTACATCAGTAAAGACCCGCAAG
CCCCCATATCCAAGCCCCATCACAACTCGCAAAAGCTGGAGGGGTTGAGA
ATTTAATGACACTCAAACAGGCATGCTCCTCGGAATACCAAGGAGCGCAA
GGTGCGTT
>OTU_269
TTAAGTTCAGCGGGTATCCCTACCTGATCCGAGGTCAAGAGTGTAAAATA
GTCTTGATGGATGCTACCGTATAAGACACAGAGCGCGATTTGTGCTGCGC
TCCGAAACTAATACGCCGGCTGCCAATTGCTTTAAGGCGAGTCCGCGCAC
AAAGGCGGGACAAACACCCAACACCAAGCAGAGCTTGAAGGTACAAATG
ACGCTCGAACAGGCATGCCCCATGGAATACCAAGGGGCGCAATGTGCGTT
>OTU_277
TTAAGTTCAGCGGGTATCCCTACCTGATCCGAGGTCAACCTGAAGTCGGC
GTCTCCGCCGACGATTTCCGGGCGCTCACCCCCCGTGTCCCGAGCGACGA
CGATGCATCACGCAGAGGAACGGGCGGCTTCCGCCGAAGACTTTGAGGCG
CGCGGCGGACCGCGACGCCCAACACCCAGCGGGGCTGGTTTGGTTGAAAT

GACGCTCGAACAGGCATGCCCCCGGAATACCAGGGGGCGCAATGTGCGT
T
>OTU_280
TTAAGTTCAGCGGGTATCCCTACCTGATCCGAGGTCAACCTTTGATGGGA
GGTTTAACGGCACAGACGCTCCTTCTCGCCAGCGAATTTGTTGCCACAAC
GCTGAGATCCAGATCGTCCAGCCGGCGACATTAAGGCGCGGGGAATCCCC
GACGCCCAAGACCAAGCAATAATTGACTTGAGTTTGTTTGATGACGCTCG
AACAGGCATGCCCCTCGGAATACCAAAGGGCGCAATGTGCGTT
>OTU_281
TTAAGTTCAGCGGGTATCCCTACCTGATCCGAGGTCAACCTGGTATAAAA
ATTCGATGGTTTGACGGCGGGAGGCCCGCCGGCTCGTCAGACGTAATCGT
AAAAGATTCTACTACGCCCAAAGCCGGTGTGACTCCGCCGAAAGCATTTG
AGGCACGCCCCCGTTTGACGGGGGGCGACGGCCCAATACCAAGCGATGCT
TGATTGGTGTAATGACGCTCGAACAGGCATGCCCCTCGGAATACCAAGGG
GCGCAATGTGCGTT
>OTU_296
TTAAGTTCAGCGGGTAGCCCTACCTGATTTGAGGTCAGATCATTGAATATG
GGGTGTTGTAAGCAGACATCGTCGGCCACGGGCGAGGCGAAACTTATCAC
GCCAAGCCATGGGTCCGACTATCCCACTGAGACCTTTAAGGCGAGCCGGC
AACAAAGCGGCAGCGCCCAAGTCCAACCCGACGGATCGTAAAACCCGGG
GGGTTGAGAGTTTCATGACACTCAAACAGGCATGCCCTCCGGAATACCAG
AGGGCGCAAGGTGCGTT
>OTU_303
TTAAGTTCAACGGGTATTCCTACCTGATCCGAGGTCAACCTTGTAAATGAT
ATGGGTTGAGACTAGAGCCGCCAGCCCCTTAAATGCGTGCTACGAGAGCT
CGTTGCATCTAGGGTCCGATGACTCCGCCATTAACTTTAGGACCCGTCTTC
AACGACGAAAGGTCCAACAGCAAGCCAGGCTTGAGGGTTGATAATGACG
CTCGAACAGGCATGCCCTTCGGAATACCAAAGGGCGCAATGTGCGTT
>OTU_305
TTAAGTTCAGCGGGTAGTCCTACCTGATTTGAGGTCAGAGCATAGAATAA
TGACCTTGCGGTCGGGTTATGAGCAGTCGTCACACCTTGACCAGACGAAA
CTTATTACGTCTTAGCCGTGGATGTTATTACCACTAACTCTTTTAAGGCGA
GCCAGCGAACTGGCAGACACCCAAGTCCAAGCCCAACACTGATCAGAGA
CCAGGAGGGGTTGATATTTCATGACACTCAAACAGGCATGCCTTTCGGAA
TACCAAAAGGCGCAAGGTGCGTT
>OTU_307
TTAAGTTCAGCGGGTAGTCCTGCCTGATTTGAGGTCAAAATGATAAGAGT
GCGCTCGGAGCGCACACCACGGGCAAGCCTAGGCTGGCCGACGAGGGCT
GTACAACGTCGGGGCCGTGAGACGGCTGCCGTTGGGCGCTGGCGACAGA
GCGCTGCATTTCGGCTCGCGACGTCGTCGGTTCCTCGCGAGAGGGGCCCG
AGTCGGTCGGAGTGGGAGCCATGATCCAAGCCGCCGCTTCTCGCAAGAGA
GGCGGGGTTGAGAGGGGAGCGCGACACTCAAACAGGCATGCTCCGCGGA
GTACCACGGAGCGCAAGGTGCGTT

Supplementary Table 2
Putative taxonomic affiliation of core dataset with 45 fungal OTUs. Fungal OTUs with 10 or more reads and their putative taxonomic assignments and accession numbers based on BLAST searches, number of reads, proportion of reads, and frequency (number of samples where each OTU was detected), represented by each OTU. Putative species level taxonomic affiliation is defined as 98-100% sequence similiarity, genus 94-97%, and order 80-93%.

OTU ID	Phylum	Subphylum	Class	Order	Accession number of closest match	Putative taxonomic assignment	% similarity	Number of reads	Proportion of reads	Frequency
OTU_3	Ascomycota	Pezizomycotina	Dothideomycetes	Venturiales	HQ599579.2	*Fusicladium peltigericola*	100	1324	0.20	55
OTU_3	Ascomycota	Pezizomycotina	Dothideomycetes	Venturiales	KF793774.1	*Venturia ditricha*	100	1324	0.20	55
OTU_5	Ascomycota	Pezizomycotina	Dothideomycetes			Dothideomycetes sp	75	878	0.13	55
OTU_6	Ascomycota	Pezizomycotina	Sordariomycetes	Hypocreales		Hypocreales sp	91	680	0.10	54
OTU_1	Ascomycota	Pezizomycotina	Leotiomycetes	Helotiales		Helotiales sp	89	570	0.08	51
OTU_10	Ascomycota	Pezizomycotina	Leotiomycetes	Helotiales		Helotiales sp	81	503	0.07	52
OTU_7	Ascomycota	Pezizomycotina	Dothideomycetes	Dothideales		Dothideales sp	83	385	0.06	48
OTU_4	Fungus					Fungus sp	83	257	0.04	50
OTU_2	Ascomycota	Taphrinomycotina	Taphrinomycetes	Taphrinales		Taphrinales sp	87	237	0.04	49
OTU_12	Basidiomycota	Agaricomycotina	Tremellomycetes	Tremellales		Tremellales sp	82	229	0.03	42
OTU_8	Basidiomycota	Pucciniomycotina	Pucciniomycetes	Pucciniales	GU049437.1	*Chrysomyxa ledi*	100	210	0.03	9
OTU_11	Ascomycota	Pezizomycotina	Arthoniomycetes	Lichenostigmatales		Lichenostigmatales sp	83	180	0.03	43
OTU_9	Ascomycota	Pezizomycotina	Eurotiomycetes	Chaetothyriales		Chaetothyriales sp	92	145	0.02	33
OTU_17	Basidiomycota	Agaricomycotina	Tremellomycetes			Tremellomycetes sp	90	122	0.02	33
OTU_13	Ascomycota	Pezizomycotina	Dothideomycetes	Dothideales	KF646090.1	*Coniozyma sp*	100	95	0.01	27
OTU_18	Ascomycota	Pezizomycotina	Lecanoromycetes	Pertusariales		Pertusariales sp	82	69	0.01	25
OTU_14	Basidiomycota	Agaricomycotina	Tremellomycetes	Filobasidiales		Filobasidiales sp	80	64	0.01	24
OTU_16	Ascomycota	Pezizomycotina	Dothideomycetes	Botryosphaeriales		Botryosphaeriales sp	81	64	0.01	28
OTU_26	Ascomycota	Pezizomycotina	Dothideomycetes			Dothideomycetes sp	79	59	0.01	22
OTU_15	Ascomycota	Pezizomycotina	Dothideomycetes	Capnodiales		*Cladosporium sp*	100	58	0.01	27
OTU_19	Ascomycota	Pezizomycotina	Dothideomycetes	Botryosphaeriales		Botryosphaeriales sp	80	49	0.01	26
OTU_20	Ascomycota	Pezizomycotina	Eurotiomycetes	Chaetothyriales		Chaetothyriales sp	85	43	0.01	19
OTU_21	Basidiomycota	Ustilaginomycotina	Exobasidiomycetes	Exobasidiales		Exobasidiales sp	93	41	0.01	16
OTU_34	Ascomycota	Pezizomycotina	Eurotiomycetes			Eurotiomycetes sp	79	37	0.01	21
OTU_22	Ascomycota	Pezizomycotina				Ascomycota sp	100	31	0.00	12
OTU_33	Ascomycota	Pezizomycotina	Dothideomycetes	Botryosphaeriales		Botryosphaeriales sp	82	27	0.00	14
OTU_24	Ascomycota	Taphrinomycotina	Taphrinomycetes	Taphrinales		Taphrinales sp	87	27	0.00	17
OTU_23	Basidiomycota	Agaricomycotina	Tremellomycetes			Cryptococcus sp	95	25	0.00	12
OTU_37	Ascomycota	Pezizomycotina	Lecanoromycetes	Trapeliales		Trapeliales sp	83	25	0.00	14
OTU_29	Ascomycota	Pezizomycotina	Dothideomycetes			Dothideomycetes sp	92	25	0.00	13
OTU_30	Ascomycota	Pezizomycotina	Dothideomycetes	Dothideales	GQ412724.1	*Sydowia polyspora*	100	24	0.00	9
OTU_47	Ascomycota	Pezizomycotina	Eurotiomycetes	Chaetothyriales		Chaetothyriales sp	84	23	0.00	10
OTU_36	Ascomycota	Pezizomycotina	Dothideomycetes	Capnodiales	EF394834.1	*Mycosphaerella excentrica*	99	22	0.00	14
OTU_39	Ascomycota	Pezizomycotina	Dothideomycetes	Pleosporales		*Alternaria sp*	100	22	0.00	8
OTU_42	Ascomycota	Pezizomycotina	Dothideomycetes	Botryosphaeriales		Botryosphaeriales sp	82	17	0.00	9
OTU_41	Ascomycota	Pezizomycotina	Dothideomycetes	Myriangiales		Myriangiales sp	86	17	0.00	10
OTU_48	Ascomycota	Pezizomycotina	Eurotiomycetes	Chaetothyriales		Ceramothyrium sp	97	16	0.00	11
OTU_35	Ascomycota	Pezizomycotina	Leotiomycetes	Helotiales		Helotiales sp	85	14	0.00	1
OTU_57	Basidiomycota	Pucciniomycotina	Microbotryomycetes	Sporidiobolales	AY015436.1	*Sporobolomyces ruberrimus*	99	14	0.00	5
OTU_43	Ascomycota	Pezizomycotina	Dothideomycetes			Dothideomycetes sp	77	13	0.00	8
OTU_46	Basidiomycota	Ustilaginomycotina	Exobasidiomycetes	Exobasidiales		Exobasidiales sp	92	13	0.00	8

OTU ID	Phylum	Subphylum	Class	Order	Accession number of closest match	Putative taxonomic assignment	% similarity	Number of reads	Proportion of reads	Frequency
OTU_44	Basidiomycota	Agaricomycotina	Agaricomycetes	Atheliales	JQ711930.1	*Piloderma sphaerosporum*	100	13	0.00	1
OTU_68	Ascomycota	Pezizomycotina	Arthoniomycetes	Lichenostigmatales		Lichenostigmatales sp	80	12	0.00	2
OTU_45	Basidiomycota	Ustilaginomycotina	Exobasidiomycetes	Exobasidiales	KR262418.1	*Exobasidium maculosum*	100	12	0.00	5
OTU_58	Ascomycota	Pezizomycotina	Sordariomycetes	Diaporthales	FR715997.1	*Sirococcus sp*	99	11	0.00	6
OTU_59	Ascomycota	Pezizomycotina	Dothideomycetes	Myriangiales		Myriangiales sp	86	10	0.00	5
